# Association Studies in *Populus tomentosa* Reveal the Genetic Interactions of *Pto-MIR156c* and Its Targets in Wood Formation

**DOI:** 10.3389/fpls.2016.01159

**Published:** 2016-08-03

**Authors:** Mingyang Quan, Qingshi Wang, Souksamone Phangthavong, Xiaohui Yang, Yuepeng Song, Qingzhang Du, Deqiang Zhang

**Affiliations:** ^1^National Engineering Laboratory for Tree Breeding, College of Biological Sciences and Technology, Beijing Forestry UniversityBeijing, China; ^2^Key Laboratory of Genetics and Breeding in Forest Trees and Ornamental Plants, Ministry of Education, College of Biological Sciences and Technology, Beijing Forestry UniversityBeijing, China

**Keywords:** association genetics, *Pto-miR156c*, miRNA-mRNA, interaction, epistasis, wood formation

## Abstract

MicroRNAs (miRNAs) regulate gene expression in many biological processes, but the significance of the interaction between a miRNA and its targets in perennial trees remains largely unknown. Here, we employed transcript profiling and association studies in *Populus tomentosa* (*Pto*) to decipher the effect of genetic variation and interactions between *Pto-miR156c* and its potential targets (*Pto-SPL15, Pto-SPL20*, and *Pto-SPL25*) in 435 unrelated individuals from a natural population of *P. tomentosa*. Single-SNP (single-nucleotide polymorphism) based association studies with analysis of the underlying additive and dominant effects identified 69 significant associations (*P* < 0.01), representing 51 common SNPs (minor allele frequency > 0.05) from *Pto-MIR156c* and its three potential targets, with six wood and growth traits, revealing their common roles in wood formation. Epistasis analysis uncovered 129 significant SNP-SNP associations with ten traits, indicating the potential genetic interactions of *Pto-MIR156c* and its three putative targets. Interestingly, expression analysis in stem (phloem, cambium, and xylem) revealed that *Pto-miR156c* expression showed strong negative correlations with *Pto-SPL20* (*r* = −0.90, *P* < 0.01) and *Pto-SPL25* (*r* = −0.65, *P* < 0.01), and a positive correlation with *Pto-SPL15* (*r* = 0.40, *P* < 0.01), which also indicated the putative interactions of *Pto-miR156c* and its potential targets and their common roles in wood formation. Thus, our study provided an alternative approach to decipher the interaction between miRNAs and their targets and to dissect the genetic architecture of complex traits in trees.

## Introduction

MicroRNAs (miRNAs)are small non-coding RNAs (20–24 nt) that function in gene regulation as sequence-specific regulators, via post-transcriptional mRNA cleavage or inhibition of gene expression in eukaryotes (Voinnet, 2009). In plants, miRNAs have influential roles in development (Rubio-Somoza and Weigel, 2011) and resistance to stresses such as drought and salinity (Frazier et al., 2011; Kruszka et al., 2012). Thus, studying miRNAs can provide important information on the mechanisms of morphological and physiological changes in response to the changeable environment in plants and for the genetic improvement of crops and forest trees (Kruszka et al., 2012; Zhou and Luo, 2013).

With the development of high-throughput sequencing technologies, many miRNAs have been identified in a range of plant species (Kozomara and Griffiths-Jones, 2014). Of these, microRNA-156 (miR156) has been well characterized in a number of plants, such as *Arabidopsis thaliana* (Wu and Poethig, 2006) and maize (*Zea mays*) (Mica et al., 2006). In *Arabidopsis* and maize, miR156 functions in shoot maturation during the juvenile to adult transition, and is highly expressed in early shoot development but decreases after this stage. Also, the juvenile to adult phase was delayed when miR156 was overexpressed (Wu and Poethig, 2006; Chuck et al., 2007). The prolonged vegetative phase caused by overexpression of miR156 mainly depends on negative regulation of the expression of *SQUAMOSA PROMOTER-BINDING PROTEIN-LIKE* genes (*SPLs*). The *SPLs* encode a plant-specific transcription factor family that plays pivotal roles throughout different stages of plant development (Birkenbihl et al., 2005); for example, *AtSPL2, AtSPL10*, and *AtSPL11* function in morphological regulation of cauline leaves and flowers during the *Arabidopsis* reproductive phase (Shikata et al., 2009).

In addition to the regulation of phase transition, the *miR156-SPL* network also functions in root development, nodulation, and stress tolerance in plants (Xie et al., 2012; Cui et al., 2014; Aung et al., 2015). For example, in *Arabidopsis* and rice (*Oryza sativa*), miR156 was highly expressed under salt and drought treatment, which reduced the expression of *SPL9*, thus increasing the expression of *DFR (DIHYDROFLAVONOL-4-REDUCTASE)* and *PAP1 (PRODUCTION OF ANTHOCYANIN PIGMENT 1)* to reinforce the accumulation of anthocyanin, leading to enhanced stress tolerance of plants (Cui et al., 2014). Over-expressing miR156 in *Populus canadensis* also prolonged the vegetative phase of trees by down-regulating the miR156 target genes *SPL3* and *SPL9*, causing phenotypic changes in leaf morphology and internode length (Wang et al., 2011).

Perennial trees have large sizes and long growth cycles; they need to adapt to changeable environmental conditions over many years. Trees also provide timber resources and many ecosystem services (Neale and Kremer, 2011). The dissection of the genetic regulatory networks for traits with complex genetic architecture, such as tree growth and wood properties, may inform efforts to improve economically and ecologically important properties. However, the interactions of the components of this system and the effect of genetic variation of the *miR156-SPL* regulatory system on tree growth and wood properties remain unclear. *P. trichocarpa* has 28 *SPL* genes that show diverse expression patterns in different tissues and organs, indicating that they may function in different biological processes in trees (Li and Lu, 2014). The regulatory mechanisms and expression patterns of the *miR156-SPL* module in tree growth and wood formation remain unknown.

In perennial woody plants, a few studies have used transgenic methods to examine the roles of miRNAs. For example, Lu et al. (2013) used transgenic *P. trichocarpa* lines over-expressing miR397 to show that miR397a negatively regulates *laccase* in lignin biosynthesis. However, trees have long generation times and lack characterized mutants, which hinders transgenic approaches aiming to clarify the functions of miRNA-mRNA interactions. However, single nucleotide polymorphism (SNP)-based association mapping provides an alternative, feasible strategy for deciphering the natural allelic variation of genes responsible for specific traits in trees (Sexton et al., 2012). For example, candidate gene-based association studies have identified several major SNPs within candidate genes associated with tree growth and wood property traits in *Eucalyptus nitens* and *Populus* (Thumma et al., 2005; Tian et al., 2012). Moreover, SNP-based association studies in humans identified SNPs in miRNA genes associated with diseases; also, SNPs in miRNA genes could alter miRNA secondary structure and influence miRNA expression levels (Duan et al., 2007; Rawlings-Goss et al., 2014).

Recently, SNP-based association mapping has emerged as a feasible way to exploring the genetic effect of miRNA-mRNA interactions on phenotypic variation. For instance, Yang et al. (2015) identified SNPs in the *Pto-MIR530a* gene and its target and evaluated their interactions and effects on tree growth and wood properties in *P. tomentosa*. In addition, epistasis modeling can detect the mutual effects of SNPs in multiple genes, providing the evidence to evaluate the complex relationships among genes (Roguev et al., 2008; Du et al., 2015), thus identifying the miRNA-mRNA interaction network that affects phenotypic variation.

In *P. trichocarpa*, the miR156 family consists of 12 members, and *Ptc-MIR156c* exhibited high abundance in xylem (Kozomara and Griffiths-Jones, 2014), indicating that miR156c might function in wood formation in trees. Here, in *P. tomentosa* (*Pto*), a genetically close species to *P. trichocarpa* (*Ptc*), we first measured the expression levels of 12 members in *Pto-MIR156* family, which showed that *Pto-MIR156c* expressed high in vascular tissue (developing xylem, mature xylem, cambium, and phloem), suggesting that this miRNA might play vital roles in the regulation of wood formation in trees. We further cloned the *Pto-MIR156c* gene and identified *Pto-SPL15, Pto-SPL20*, and *Pto-SPL25* as the potential targets of *Pto-miR156c* via bioinformatics prediction and degradome sequencing. We test the expression levels of *Pto-MIR156c* and mature *miR156*, which revealed a significant positive correlation. Then, we deciphered the genetic variations and interactions of *Pto-miR156c* and its putative targets associated with tree growth and wood property traits, using SNP-based association mapping with underlying additive, dominant, and epistasis effects in a natural population of 435 unrelated individuals of *P. tomentosa*. Single-SNP-based association mapping identified SNPs within *Pto-MIR156c* and its putative targets significantly associated with tree growth and wood properties. Combined with expression pattern analysis of *Pto-MIR156c* and its potential targets, this reveals their common roles in wood formation. We also detected the SNPs in the pre-miRNA (precursor miRNA) region of *Pto-MIR156c* that affected the stability of its secondary structure. In addition, epistasis modeling provided evidence for the interaction of *Pto-miR156c* and its potential targets. Thus, our study provided a better understanding of *miR156-SPL* regulatory network in tree growth and wood formation and raised an alternative method for dissecting the genetic variation and interactions of miRNAs and mRNAs in population genetics of trees.

## Materials and methods

### Population and phenotypic data

The association population was composed of 435 unrelated individuals, selected from 1047 individuals of the *P. tomentosa* collection established in Guan Xian Country (Shandong province, China, 36°23′N, 115°47′E) in 1982, using a randomized complete block design approach with three clonal replications, covering almost all the natural distribution of *P. tomentosa*, i.e., the southern, northwestern, and northeastern regions of China (30–40°N, 105–125°E) (Du et al., 2012). In addition, 43 unrelated individuals, representing almost the entire range of native *P. tomentosa*, selected from the association population were used to identify SNPs within candidate genes.

Three growth traits and seven wood property traits were measured with at least three replications per genotype and these data were used for association analysis. The three growth traits were: tree height (H, m), diameter at breast height (DBH, cm), and stem volume (V, m^3^). The seven wood properties were: holocellulose content (HC, %), α-cellulose content (CC, %), lignin content (LC, %), hemicellulose content (HEC, %), fiber width (FW, μm), fiber length (FL, mm), and microfibril angle (MFA, °). The detailed measurement methods and correlations of these phenotypic data for 435 genotypes were described previously (Du et al., 2014).

### Identification of *Pto-MIR156c* and three potential targets of *Pto-miR156c*

The primary sequence of *Pto-MIR156c* was cloned based on the sequence of *Ptc-MIR156c* from *P*. *trichocarpa* (Kozomara and Griffiths-Jones, 2014), containing the pre-miRNA sequence and 300 bp flanking regions on each side of the pre-miRNA region, using gene-specific primers. Two screening methods, psRNATarget prediction and degradome sequencing, were used to determine the target genes of *Pto-miR156c*. psRNATarget (http://plantgrn.noble.org/psRNATarget/) was used to predict the targets of *Pto-miR156c* using 3000 complementary DNA (cDNA) sequences from a mature xylem cDNA library of *P. tomentosa*. This cDNA library was constructed with the Superscript λ System (Life Technology, Carlsbad, CA, USA) with the manufacturer's instructions and consisted of 5.0 × 10^6^ pfu with an insert size of 1.0–4.0 kb (Li et al., 2009). Then, random end-sequencing of 3000 cDNA clones from this cDNA library were used for further analysis. In addition, we employed *P. tomentosa* degradome sequencing (described below), a high-throughput sequencing method which is widely used for verifying the regulatory relationship of miRNAs and mRNAs, to identify the miRNA cleavage sites. After filtering by these two screening approaches, the target genes of *Pto-miR156c* were determined and the full-length cDNAs of target genes were isolated from the 3000 cDNA clones.

### Degradome sequencing

Total RNA of six tissues (leaf, shoot apex, phloem, cambium, developing xylem, and mature xylem) was extracted using the Plant Qiagen RNeasy kit (Qiagen China, Shanghai) following the manufacturer's instructions. Additional, on-column DNase digestions were applied using RNase-Free DNase Set (Qiagen) during the RNA purification and the RNA integrity was confirmed on an agarose gel. The total RNA samples from the six tissues were pooled together in equal amounts, which were used to construct degradome libraries according to the methods described previously (Shamimuzzaman and Vodkin, 2012). Briefly, a 5′ RNA adapter, which possesses a free 5′-monophosphate at the 3′ terminus, was added to the cleavage products using T4 RNA ligase (Ambion). Then the ligated products were purified and reverse transcribed using an oligo dT primer by SuperScript II RT (Invitrogen). The resulting cDNA was amplified for 6 cycles (94°C for 30 s, 60°C for 20 s, and 72°C for 3 min), and then the PCR products were digested with MmeI and ligated to a 3′ double adapter. Finally, the ligation products were amplified, gel-purified, and used for sequencing-by-synthesis with the Illumina HiSeq2000. The CleaveLand pipeline (Addo-Quaye et al., 2009) was used to analyze the miRNA cleavage sites based on the *P. trichocarpa* genome transcripts (V3.0) (SRX1447192).

### Tissue-specific expression analysis

Seven fresh tissues, including young leaf, old leaf, shoot apex, phloem, cambium, developing xylem, and mature xylem, were sampled from 1-year-old *P. tomentosa* clone “LM50.” Total RNAs from these fresh tissues were extracted using the methods described above. Then, cDNAs for the seven tissues were synthesized with the Plant Qiagen RNasey Kit, and these cDNAs were used for testing the tissue-specific expression of *Pto-MIR156c* and the three potential targets of *miR156c*. Small RNAs (< 200 nt) were isolated via the mirVana miRNA Isolation Kit (Ambion, USA) following the manufacturer's instructions, which were used for testing the expression levels of mature miRNA. Then, poly(A) tails were added to 3′ end of the small RNAs via poly(A) polymerase (Ambion), and the polyadenylated small RNAs were revised transcribed with oligo(dT) adapter (Ambion).

Reverse transcription quantitative PCR (RT-qPCR) was performed on a 7500 Fast Real-Time PCR System using the SYBR Premix Ex Taq (TaKaRa, Dalian, China). The gene-specific primers (Table S1) designed by Primer Express 3.0 software (Applied Biosystems) were used to determine the expression levels of 12 members (a-l) in *Pto-MIR156* gene family, mature *miR156*, and its putative targets. All reactions were performed with triplicate technical and triplicate biological repetitions, with *Actin* (EF145577) as the internal control. The data were analyzed with the Opincon Monitor Analysis software 3.1 tool and the melting curve was used to check the specificity of the amplified fragments. The conditions for PCR were: 94°C for 5 min; 40 cycles of 94°C for 30 s, 58°C for 30 s, and 72°C for 30 s; and a final with 70–95°C for the melting curves, which were used to confirm the specificity of the amplification.

### SNP discovery and genotyping

To identify SNPs, not including Indels (insertions/deletions), in *Pto-MIR156c* and its three potential targets (i.e., *Pto-SPL15, Pto-SPL20*, and *Pto-SPL25*), the full-length genomic regions of the four genes were sequenced, using gene-specific primers designed based on the cDNA clones of the three genes, and analyzed in 43 unrelated individuals randomly selected from the *P. tomentosa* association population. The clone and PCR amplification procedures were according to Zhang et al. (2011). The BigDye Terminator Cycle Sequencing kit (version 3.1, Applied Biosystems, Beijing, China) and the 4300 DNA Analyzer (Li-Cor Biosciences, Lincoln, NE, USA) were used for sequencing of candidate genes. Using the DNeasy Plant Mini Kit (Qiagen China, Shanghai), genomic DNA was extracted from fresh leaf tissue of the 43 *P. tomentosa* individuals and used as DNA amplification templates.

Sequences of *Pto-MIR156c, Pto-SPL15, Pto-SPL20*, and *Pto-SPL25* in the 43 individuals have been deposited in GenBank under the accession numbers KX080106—KX080148, KX079977—KX080019, KX080020—KX080062, and KX080063—KX080105, respectively. MEGA 5.0 (Tamura et al., 2011) was used for sequence alignment and SNP identification, and DnaSP 5.10 (Librado and Rozas, 2009) was employed to evaluate the nucleotide diversity. Then, all the common SNPs (minor allele frequency (MAF) > 0.05) identified were genotyped in the 435 individuals from the association population using the Beckman Coulter (Franklin Lakes, NJ, USA) sequencing system.

### Data analysis

#### Linkage disequilibrium (LD) analysis

The squared correlation of allele frequencies (*r*^2^) between each pair of common SNPs (MAF > 0.05) within *Pto-MIR156c, Pto-SPL15, Pto-SPL20*, and *Pto-SPL25*, was used to assess the extent of LD using TASSEL v. 5.0 (Bradbury et al., 2007). The decay of LD with distance in base pairs (bp) between SNPs was evaluated by non-linear regression with 10^5^ permutations of the data (Remington et al., 2001), and singletons were excluded in LD analyses.

#### Single SNP-based association analysis

The mixed linear model (MLM) in TASSEL v.5.0 (Bradbury et al., 2007) was used for single SNP-based association in the association population. The MLM was: y = μ + Qv + Zu + e, with y denoting a vector of phenotypic observations, μ denoting the intercepts vector, v denoting a vector for population effects, u denoting the vector of random polygenic background effects, e denoting random experimental error, Q matrixes defining the population structure, and Z being the matrixes relating y to u. In addition, we used the estimated membership probability (Q) and pairwise kinship (K) to evaluate the population structure and relatedness, respectively, among individuals for marker-trait associations. The K matrix was assessed by SPAGeDi 1.3 (Hardy and Vekemans, 2002) based on 20 species-specific SSR markers (Du et al., 2012), and the Q matrix was evaluated based on significant subpopulations (*k* = 3) according to the statistical model described by Evanno et al. (2005) via STRUCTURE V.2.3.4 (Patterson et al., 2006). Finally, the positive false discovery rate (FDR) was used for correcting multiple tests, using QVALUE running in R (Storey and Tibshirani, 2003).

#### Haplotype-based association analysis

Haplotype trend regression (HTR) analysis was conducted to estimate the haplotype frequencies from the genotype data on a three-marker sliding window and test the haplotype-based associations (Zaykin et al., 2002). The significance of haplotype-based associations was based on 10^4^ permutation tests, and singleton alleles and haplotypes with frequencies less than 0.05 were discarded in this analysis.

#### Multi-SNP based epistasis analysis

A multifactor dimensionality reduction (MDR) algorithm (Hahn et al., 2003), which processed high-dimensionality genetic data into a single dimension so that the interactions could be detected in a relatively small set, was used to dissect the epistatic effects (non-additive interactions) among SNPs. In MDR 3.0.2, the ReliefF algorithm, which improves the reliability of probability approximation, filtered all unlinked SNPs (*r*^2^ < 0.1 or different genes), and produced the five most-significant SNPs for each trait. In addition, the genetic effects of significant SNP-SNP pairs were evaluated by information gain (IG), calculated by entropy-based measure (Moore et al., 2006).

### Transcript analysis of SNP genotypes

RT-qPCR was conducted to test the effect of different genotypes of SNPs from *Pto-MIR156c* and its potential targets on transcript abundances in mature xylem. Ten individuals for each genotype of every SNP were randomly selected from the association population, and the RT-qPCR was performed as described above. Differential expression across different genotypic pairs was evaluated by ANOVA.

## Results

### Identification of *Pto-MIR156c* and three potential targets of *Pto-miR156c*

**Figure 1:**
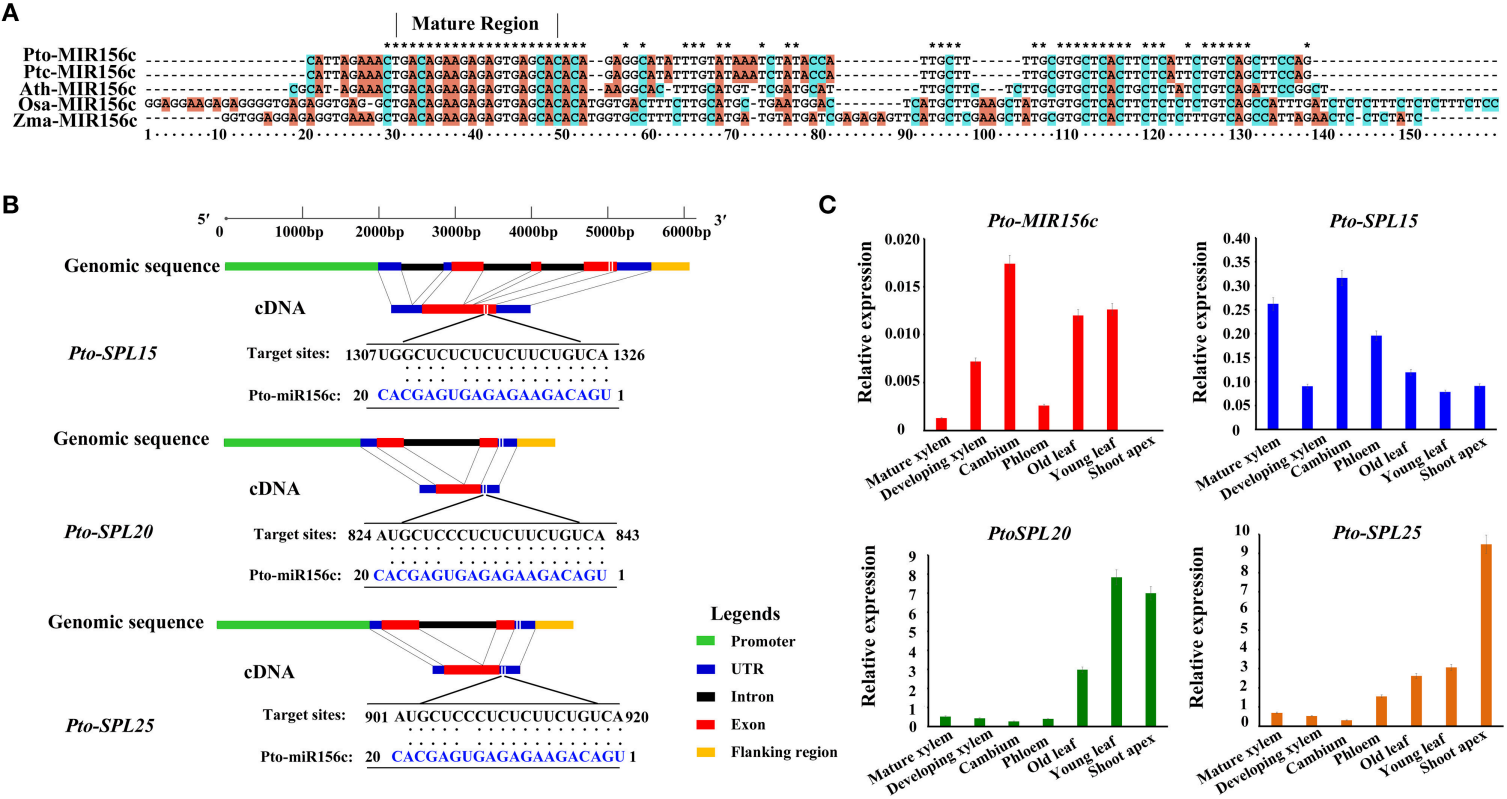
**Characterization of Pto-miR156c and its three potential targets and expression analysis of ***Pto-MIR156c***, ***Pto-SPL15***, ***Pto-SPL20***, and ***Pto-SPL25***. (A)** The alignment result for pre-miRNA region of MIR156c gene among the species of *P. tomentosa* (*Pto*), *P. trichocarpa* (*Ptc*), *A. thaliana* (*Ath*), *O. sativa* (*Osa*), and *Z. mays* (*Zma*). The asterisks indicated the nucleotides in the five species are conserved. **(B)** The gene structures of *Pto-SPL15, Pto-SPL20*, and *Pto-SPL25*, and the *Pto-miR156c* target sites determined by degradome sequencing. **(C)** The relative expression levels (arbitrary units normalized to control) of Pto-MIR156c and its three potential targets in seven tissues, including mature xylem, developing xylem, cambium, phloem, old leaf, young leaf, and shoot apex, conducted by RT-qPCR with Actin as the internal control.

To examine the expression patterns of *Pto-MIR156* gene family in different tissues and organs, we first measured the expression abundance of 12 members in *Pto-MIR156* family, which showed that *Pto-MIR156c* exhibited high abundance in vascular tissue (developing xylem, mature xylem, cambium, and phloem), indicating that *Pto-miR156c* might function in wood formation of trees (Figure S1). Then, to identify SNPs in *Pto-MIR156c*, we cloned the primary sequence of *Pto-MIR156c* gene based on the sequence of the *P. trichocarpa* homolog *Ptc-MIR156c* in miRbase. The sequencing yielded the 700-bp genomic sequence of the *Pto-MIR156c* primary transcript, including 100 bp of pre-miRNA sequence, the 20-bp mature miRNA, and 300 bp of flanking sequence on each side of the pre-miRNA region. Prediction of the secondary structure for the *Pto-MIR156c* pre-miRNA sequence by RNAfold analysis revealed a typical hairpin structure, confirming that *Pto-miR156c* is a miRNA. Comparison of the *Pto-MIR156c* pre-miRNA sequence with homologous miRNAs in *P. trichocarpa, Oryza sativa, Zea mays*, and *Arabidopsis thaliana* revealed that the mature region of these miRNAs were completely conserved, despite the diverse degrees of similarity (52.32–100%) in the pre-miRNA region (Figure 1A).

We further used the 20-nt mature sequence and psRNATarget to identify 56 potential target genes of *Pto-miR156c*, and degradome sequencing to detect the most likely cleavage sites in the three putative target genes (Figures S2–S4). After filtering with two screening methods, three full-length cDNAs, *Pto-SPL15, Pto-SPL20*, and *Pto-SPL25*, were identified as target genes of *Pto-miR156c*. The cDNA sequences of *Pto-SPL15, Pto-SPL20*, and *Pto-SPL25* isolated from a cDNA library from *P. tomentosa* mature xylem showed that *Pto-SPL15, Pto-SPL20*, and *Pto-SPL25* were 1850, 1061, and 1159 bp in length encoding proteins of 328, 196, and 242 amino acids, respectively, and these proteins contain the conserved SBP (SQUAMOSA-PROMOTER BINDING PROTEIN) domain. The encoded proteins also showed high amino acid sequence similarity to the proteins encoded by their *P. trichocarpa* homologs *Ptc-SPL15* (84.15%), *Ptc-SPL20* (97%), and *Ptc-SPL25* (74.26%) (Figure 1B).

### Expression profiles of *Pto-MIR156c* and the three potential targets of *Pto-miR156c*

To test the tissue-specific expression and expression patterns of *Pto-MIR56c* and its three putative targets, we used RT-qPCR to measure transcript abundance in seven different tissues and organs of *P. tomentosa*. The results revealed that *Pto-MIR156c* is expressed in all examined tissues except the shoot apex, and the transcripts of *Pto-SPL15, Pto-SPL20*, and *Pto-SPL25* were also present in the seven tissues and organs, with varied abundance (Figure 1C). For *Pto-MIR156c*, the expression peaked in cambium, followed by young leaf, and the lowest abundance was observed in mature xylem, except for shoot apex. *Pto-SPL15, Pto-SPL20*, and *Pto-SPL25* predominantly expressed in cambium, young leaf, and shoot apex, respectively. By contrast, *Pto-SPL15* had lower abundance in young leaf, and *Pto-SPL20* and *Pto-SPL25* were poorly expressed in cambium. Then, we measured the expression levels of mature *miR156* in the seven tissues and organs, which showed a significant positive correlation with *Pto-MIR156c* (*r* = 0.90, *P* < 0.05) (Figure S5). Interestingly, the expression correlation analysis revealed that the expression levels of *Pto-MIR156c* and the three putative targets (*Pto-SPL15, Pto-SPL20*, and *Pto-SPL25*) exhibited weak or no correlation (*r* = 0.17, −0.04, and −0.42, respectively, *P* < 0.05) in the seven tissues and organs. However, in stem, including phloem, cambium, developing xylem, and mature xylem, the transcript levels of *Pto-MIR156c* and *Pto-SPL15* (*r* = 0.40, *P* < 0.05) showed a significant positive correlation, and *Pto-MIR156c* and the two other targets showed significant, strong negative correlations [*r*(*Pto-SPL20*) = −0.90, *r*(*Pto-SPL25*) = −0.65]. These results indicated that the miR156c-*SPL* network in *P. tomentosa* may participate in the regulation of wood formation.

### Nucleotide diversity and LD in *Pto-MIR156c* and the three potential targets of *Pto-miR156c*

To identify polymorphisms for association studies, we sequenced the genomic regions of the four genes in 43 unrelated individuals from the association population. For *Pto-MIR156c*, we detected 24 SNPs with an average density of 1/27 bp (π = 0.01139 and θ_w_ = 0.00792). The mature region had no SNPs, indicating that the mature region was the most conserved, and the pre-miRNA region had two SNPs, a frequency of 1/50 bp (π = 0.01023 and θ_w_ = 0.00468). In addition, we identified 370 SNPs in *Pto-SPL15, Pto-SPL20*, and *Pto-SPL25* with frequencies of 1/37, 1/31, 1/71, respectively (Table 1 and Table S2). Within the coding regions of *Pto-SPL15, Pto-SPL20*, and *Pto-SPL25*, the average synonymous diversity (*d*_*S*_) was higher than the non-synonymous diversity (*d*_*N*_) with the *d*_*N*_ /*d*_*S*_ ratio of < 1 (0.97, 0.92, and 0.35, respectively) for all exons, suggesting that the non-synonymous sites in the exon region experienced purifying selection.

**Table 1:** **Summary of single nucleotide polymorphisms of ***Pto-MIR156c*** and the three potential targets of ***Pto-miR156c*****.

**Gene**	**Region**	**Length (bp)**	**Number of polymorphic sites**	**Number of common SNPs**	**Percentage polymorphisms (%)**	**Nucleotide diversity**	
						**π**	**θw**
***Pto-MIR156c***							
	Flanking region	600	22	15	3.67	0.01159	0.00847
	Pre-miRNA region	100	2	2	2.00	0.01023	0.00462
	Mature region	20	0	0	0.00	0	0
	Total	700	24	17	3.43	0.01139	0.00792
***Pto-SPL15***							
	Synonymous	221.11	9	8	4.07	0.00941	0.00941
	Non-synonymous	762.89	29	27	3.80	0.00916	0.00879
	Total silent^a^	5319.11	137	84	2.58	0.00565	0.00595
	Total^b^	6082	166	111	2.73	0.00612	0.00635
***Pto-SPL20***							
	Synonymous	127.88	4	2	3.13	0.00282	0.00723
	Non-synonymous	460.12	8	4	1.74	0.00262	0.00402
	Total silent^a^	3875.88	130	85	3.35	0.00667	0.00775
	Total^b^	4336	138	89	3.18	0.00624	0.00736
***Pto-SPL25***							
	Synonymous	163.06	4	4	2.45	0.01021	0.00567
	Non-synonymous	562.94	7	7	1.24	0.00361	0.00287
	Total silent^a^	4106.06	59	59	1.44	0.00523	0.00332
	Total^b^	4669	66	66	1.41	0.00504	0.00327

a *Total silent: synonymous sites plus polymorphic sites in non-coding regions of genes*.

b *Total: silent sites plus non-synonymous sites of genes*.

**Figure 2:**
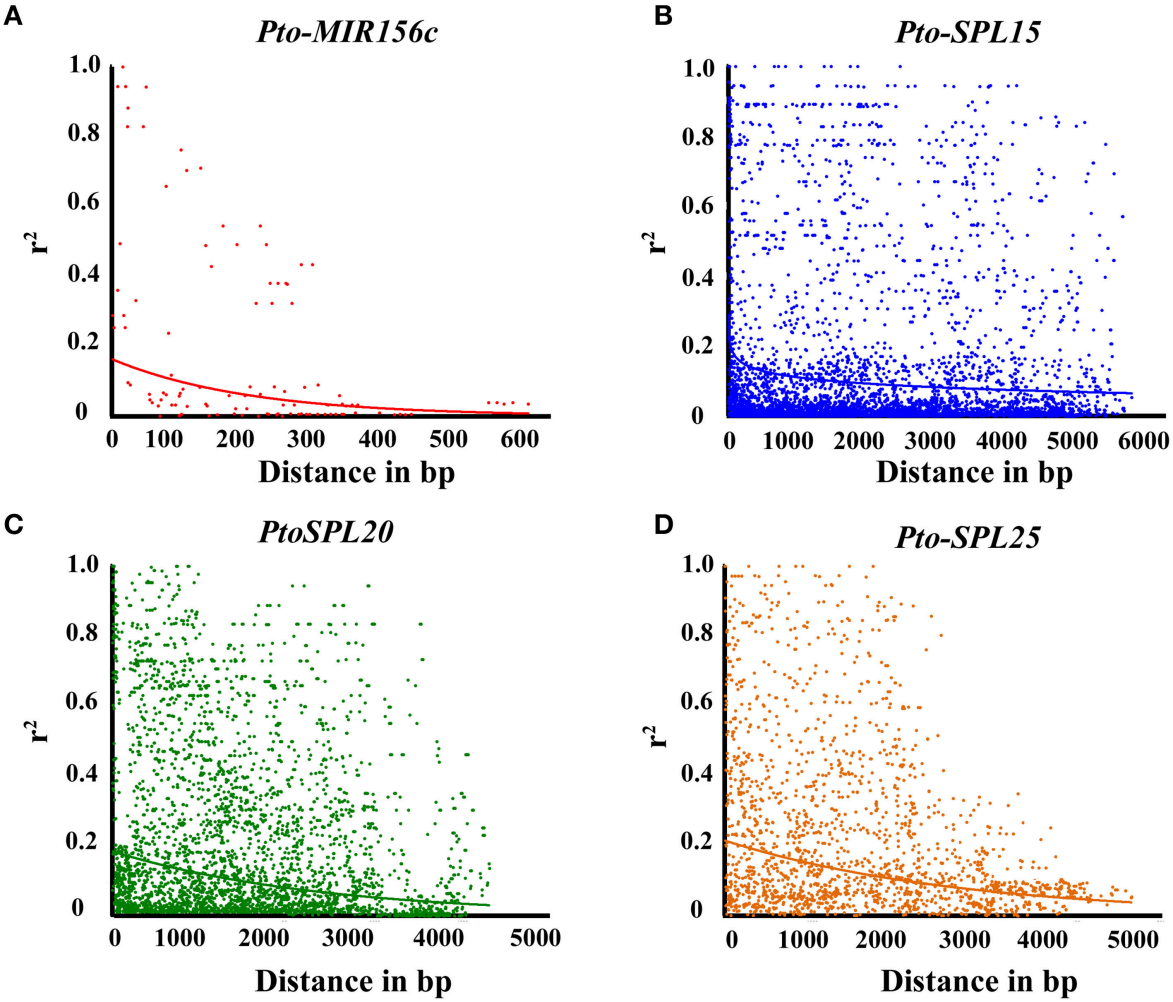
**Linkage disequilibrium within ***Pto-MIR156c*** (A), ***Pto-SPL15*** (B), ***Pto-SPL20*** (C), and ***Pto-SPL25*** (D)**. Pairwise correlations between SNPs are plotted against the physical distance between in base pairs. The curves indicate the non-linear regressions of *r*^2^ onto the physical distance in base pairs.

To perform association studies, a set of 283 common SNPs (MAF > 0.05) from *Pto-MIR156c* and the three putative targets of *Pto-miR156c* were genotyped in 435 unrelated individuals of the association population (Table 1 and Table S3), of which 17 common SNPs were found in *Pto-MIR156c*, including 2 in the pre-miRNA region and 15 in the flanking region. Of the 263 common SNPs in three target genes, 80.52% were found in non-coding regions, including the promoter (41.20%), 5′/3′ UTRs (un-translated regions, 11.61%), intron (20.60%), and flanking region (500 bp downstream of the 3′ UTR, 11.38%) of the genes (Table S2). The remaining SNPs were in coding regions with 14.23% non-synonymous changes and 5.24% synonymous changes. The *r*^2^-values of all pairwise combinations, integrated with their physical distances, were used to evaluate the overall patterns of LD for *Pto-MIR156c* and the miRNA putative targets (Figure 2). We found that LD decayed rapidly with *r*^2^, declining to 0.1 within about 100–1500 bp for each gene, indicating that LD of *Pto-MIR156c* and the potential targets did not extend to the whole genes.

### Genetic effect of allelic variation in *Pto-MIR156c* and the *Pto-miR156c* potential targets revealed by association studies

#### Single SNP-based association

**Figure 3:**
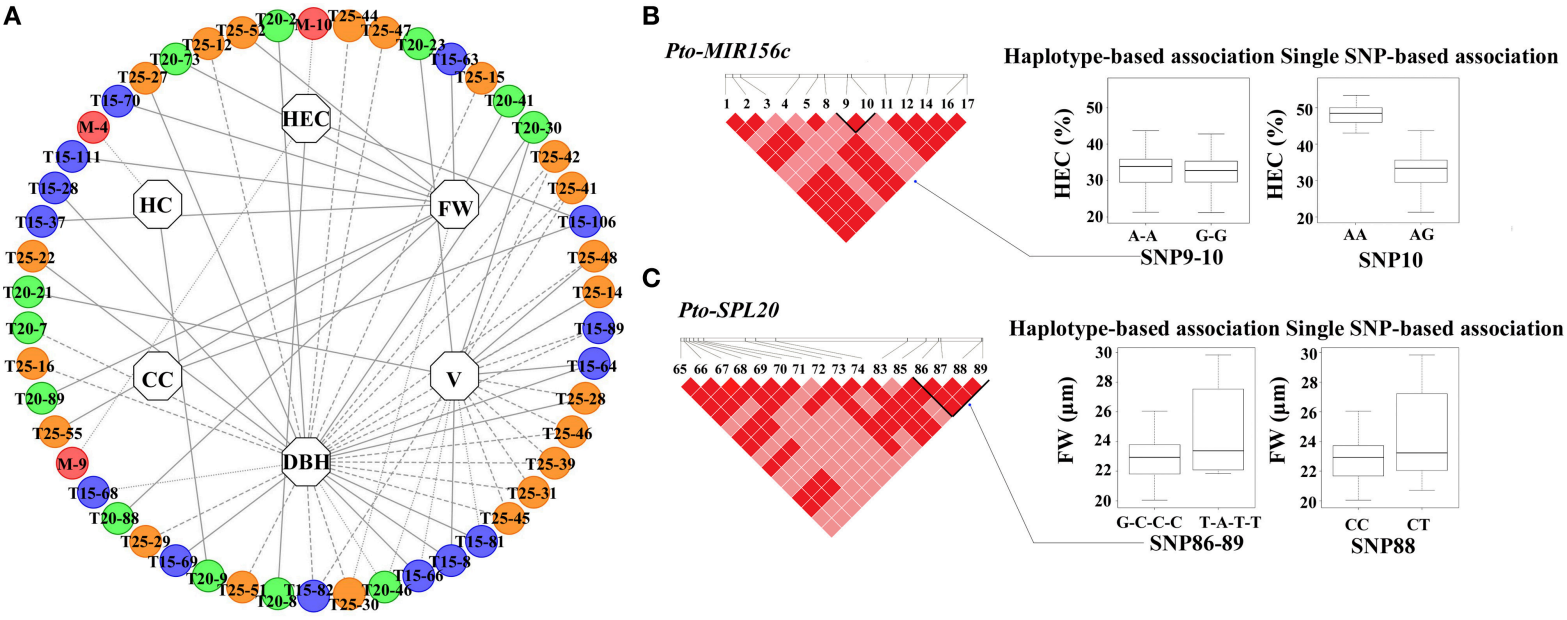
**The single SNP-based associations with additive and dominant effects and the haplotype-based associations in the association population of ***P. tomentosa***. (A)** The single SNP-based associations between significant SNPs from *Pto-MIR156c* and its three potential targets and traits. The dashed lines represent the significant associations with additive effects, the dotted lines represent the significant associations with dominant effects, and the solid lines represent the associations with both additive and dominant effects. In addition, the circles with M (red), T15 (blue), T20 (green), and T25 (orange) markers (e.g., T15-63) in the outer lane indicate the associated SNPs from *Pto-MIR156c, Pto-SPL15, Pto-SPL20*, and *Pto-SPL25*, respectively. The inner lane represents the associated traits. **(B,C)** The genotypic effect for the significant haplotypes of *Pto-MIR156c-SNP9-10* and *Pto-SPL20-SNP86-89*, along with the genotypic effect for the single locus of *Pto-MIR156c-SNP10* and *Pto-SPL20-SNP88*, which is consistent with single SNP-based association.

To investigate the genetic effects of SNPs in *Pto-MIR156c* and the three potential targets of *Pto-miR156c* on tree growth and wood properties, we used MLM in TASSEL 5.0 to conduct 2830 association tests between 283 common SNPs from *Pto-MIR156c* and the putative targets and 10 traits (H, DBH, V, HC, HEC, CC, LC, FW, FL, and MFA). In total, we detected 69 significant associations (*P* < 0.01, *Q* < 0.1) representing 51 unique SNPs in *Pto-MIR156c* and the three putative targets and six traits with 2.79–19.32% of the phenotype variance (*R*^2^) explained by each SNP (Figure 3A, Table 2, and Table S4). For *Pto-MIR156c*, three SNPs significantly associated with HC and HEC, indicating the pivotal role of *Pto-miR156c* in wood formation. *Pto-MIR156c*-SNP9 and *Pto-MIR156c*-SNP10, located in the pre-miRNA region, associated with HEC with different phenotypic contributions and *Pto-MIR156c*-SNP4 in the flanking region associated with HC. In addition, 48 SNPs from *Pto-SPL15, Pto-SPL20*, and *Pto-SPL25* significantly associated with six traits (DBH, V, FW, HEC, CC, and HC), and 16.67% of the SNPs showing associations were in coding regions (Table 2). The number of associations varied across trait categories (Table S4), with 51 for growth traits, 4 for wood composition, and 11 for wood physical properties, suggesting the vital roles of *Pto-SPL15, Pto-SPL20*, and *Pto-SPL25* in tree growth and wood formation.

**Table 2:** **Summary of significant SNPs within ***Pto-MIR156c*** and the three potential targets of ***Pto-miR156c*** associated with growth and wood properties in the association population of ***P. tomentosa*****.

**Gene**	**Number of associated SNPs**	**Number of associations**	**Traits**	**Range of additive effect**	**Range of dominant effect**	**Range of *R*^2^ (%)**
*Pto-MIR156c*	3	3	HC, HEC	–	−7.5 to −3.73	2.79–5.05
*Pto-SPL15*	14	21	DBH, V, FW, CC, HEC	0.06–31.44	−24.76 to 45.81	4.23–19.32
*Pto-SPL20*	12	14	DBH, V, FW, HC, HEC	0.62–32.05	−38.07 to 4.74	4.24–11.60
*Pto-SPL25*	21	31	DBH, V, FW	0.30–32.60	−3.20 to 3.85	4.46–16.28

Of the 69 SNP-trait associations, 86.96% showed additive effects, 57.97% showed dominant effects, and 44.93% showed combined additive and dominant effects (Figure 3A). In total, 60 significant associations were found to have an additive effect of 0.06–32.60 (Table 2). Among these, *Pto-SPL25*-SNP41 in the 5′ UTR associated with V and had the largest effect. Also, 32 SNPs within *Pto-MIR156c* and its potential targets associated with six traits with dominant effects ranging from −38.07 to 45.81, and positive and negative dominant values for half of the effects. Of these associations, *Pto-SPL15*-SNP64 in the promoter region associated with V had the largest positive dominant effect, and *Pto-SPL20*-SNP21, in an exon, caused a non-synonymous mutation of Ala to Gly, associated with V, and had the largest negative dominant effect (Table S4). Interestingly, 17 significant SNPs from *Pto-MIR156c* and the potential targets associated with more than one trait with disparate additive and/or dominant effects and phenotypic contributions to each trait (Figure 3A). In addition, each trait associated with 2-32 significant SNPs from *Pto-MIR156c* and its putative targets with different contributions to phenotypic variation, indicating that *Pto-MIR156c* and the three potential targets of *Pto-miR156c* may affect tree growth and wood properties in the same pathway.

#### Haplotype-based association

We also identified 65 common haplotypes (frequency > 0.05) from 32 high-LD blocks (*r*^2^ > 0.7, *P* < 0.001) within *Pto-MIR156c* and the target genes (Table 3 and Table S5). The number of LD blocks for each gene varied from 4 to 11, and each block contained 2–3 common haplotypes with an average of two (Table 3). Haplotype-based association, performed by HTR, detected 72 significant associations (*P* < 0.01) between 44 common haplotypes from 26 blocks within *Pto-MIR156c* and the putative targets genes, and seven traits (DBH, V, FW, FL, MFA, HEC, and CC) with *R*^2^ ranging from 0.14 to 29.47% (Table S5).

**Table 3:** **Summary of haplotype-based association analysis within ***Pto-MIR156c*** and the three potential targets of ***Pto-miR156c*** for each trait in the association population of ***P. tomentosa*****.

**Gene**	**Number of LD blocks**	**Number of common haplotypes^a^**	**Length range of haplotypes^b^**	**Number of associated haplotypes^c^**	**Associated traits**	**Range of *R*^2^ (%)**
*Pto-MIR156c*	4	8	2–5	7	DBH, FW, CC, HEC	0.15–4.35
*Pto-SPL15*	10	20	2–9	15	DBH, V, FW, FL, MFA, HEC	0.66–13.59
*Pto-SPL20*	11	23	2–6	15	DBH, V, FW, MFA,CC, HEC	1.21–7.67
*Pto-SPL25*	7	14	2–6	7	DBH, V, FW, CC, HEC	2.20–29.47

a *Common haplotype: frequency ≥ 0.05*.

b *Length range of haplotypes: one SNP as a unit*.

c *Associated haplotypes: the significant level for association with P < 0.01*.

The number of associated haplotypes for each trait ranged from 1 to 24; for example, 24 common haplotypes, including five in *Pto-MIR156c*, nine in *Pto-SPL20*, three in *Pto-SPL25*, and seven in *Pto-SPL15*, simultaneously associated with FW, with *R*^2^ ranging from 0.68 to 21.73% (Table S5). In addition, 50% of the significantly associated haplotypes were shared among traits. For instance, one haplotype (C-G-C-C) from block1 in SNP3-6 of *Pto-SPL20* associated with DBH, HEC, MFA, and V, explaining 1.30–4.79% of the phenotypic variance. Examination of haplotype association was also supported by single-SNP-based association. For example, two haplotypes (A-A and G-G) from *Pto-MIR156c*-SNP9-10 and two haplotypes (G-C-C-C and T-A-T-T) from *Pto-SPL20*-SNP86-89 associated with HEC and FW, respectively, which were strongly supported by single SNP-based associations for the same traits (Figures 3B,C).

### The interactions of *Pto-miR156c* and its three potential targets revealed by epistasis modeling

**Figure 4:**
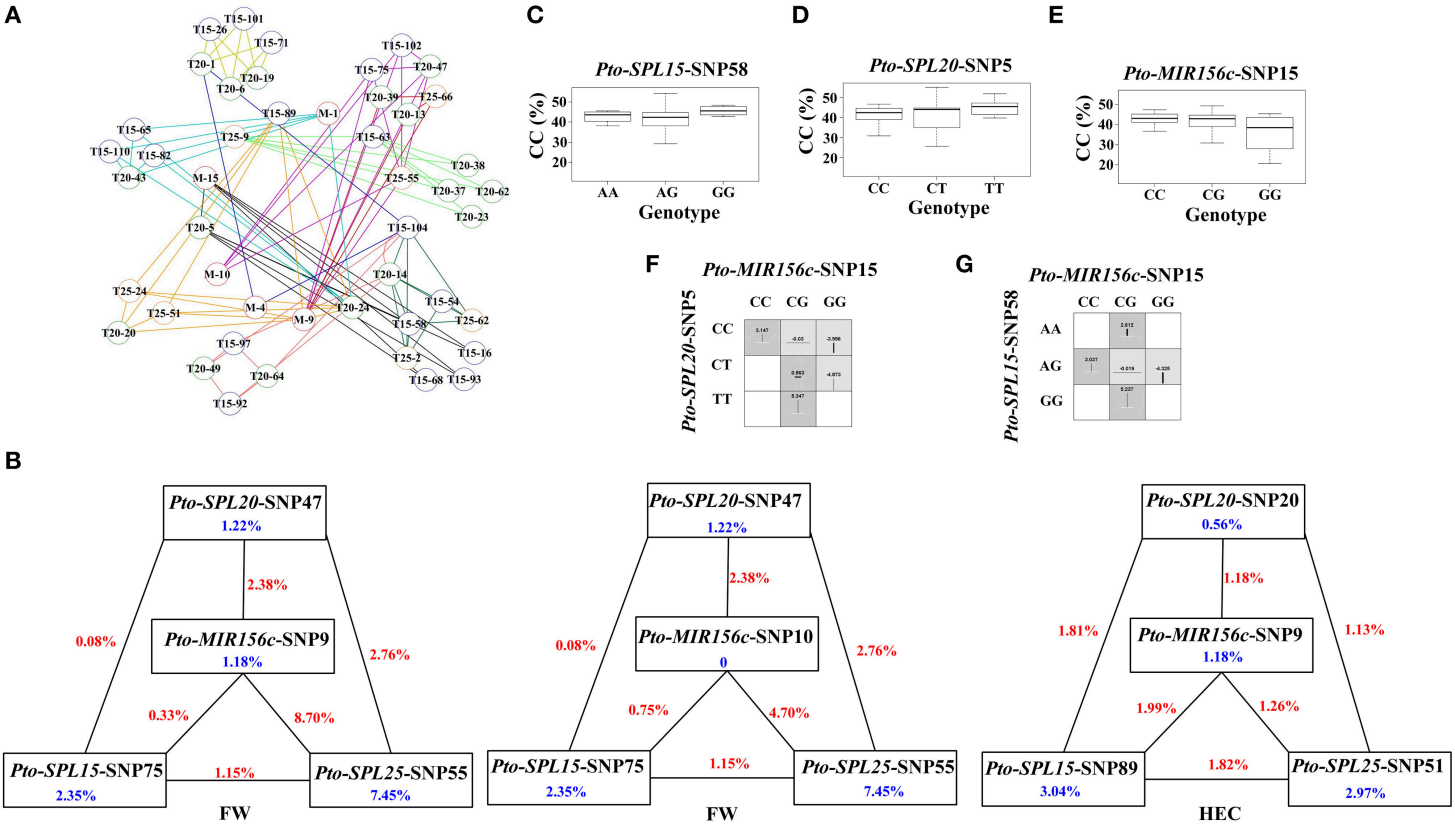
**The epistatic network within the SNPs from ***Pto-MIR156c*** and the three potential targets of ***Pto-miR156c***, and the phenotypic variations of single-locus genotypes and pairwise genotypic combinations. (A)** A structural network revealed the epistatic interactions of different loci in *Pto-MIR156c* and its three potential targets. Lines with different colors represent the different associated traits of the SNP pairs, with colors representing the different traits: yellow (height, H), red (stem diameter, DBH), pink (stem volume, V), pale green (holocellulose content, HC), orange (hemicellulose content, HEC), pale blue (lignin content, LC), black (α-cellulose content, CC), blue (fiber length, FL), purple (fiber width, FW), and deep green (microfiber angle, MFA). In addition, the circles with M (red), T15 (blue), T20 (green), and T25 (orange) markers (e.g., T15-63) indicate the associated SNPs from *Pto-MIR156c, Pto-SPL15, Pto-SPL20*, and *Pto-SPL25*, respectively. **(B)** Interaction graph for FW and HEC among SNPs in *Pto-MIR156c, Pto-SPL15, Pto-SPL20*, and *Pto-SPL25*. The blue values in boxes represent the single-marker effect, and the red values in lines indicate the pairwise epistatic effect. **(C–E)** Box plots reveale the single-locus phenotypic variation of different genotypes of three SNPs. **(F,G)** Square boxes show the pairwise phenotypic variation of different genotypic combinations from different SNP pairs.

To characterize the functional roles and interactions of *Pto-miR156c* and the three putative targets of *Pto-miR156c* in wood formation, we used MDR 3.0.2 to perform a SNP-SNP association study based on epistasis effects between *Pto-MIR156c* and the three potential targets of *Pto-miR156c*, and the 10 traits. In total, we detected 129 significant pairwise associations (*P* < 0.01, *Q* < 0.1) representing 47 unique SNPs from *Pto-MIR156c* (5), *Pto-SPL15* (18), *Pto-SPL20* (17), and *Pto-SPL25* (7) and 10 traits with single effects from 0 to 7.45% and pairwise effects from 0 to 8.7% (Figure 4A, Table 4, and Table S6). The pairwise epistatic effect was assessed by information gain (IG, ranging from −0.87 to 3.26%), of which 85.27% SNP-SNP associations behaved with negative IGs (Table 4). Of the SNP-SNP interactions, 21.71 and 49.61% represented miRNA-mRNA and mRNA-mRNA interactions, respectively, and the remaining (28.68%) consisted of SNPs within the same genes. In addition, one SNP in *Pto-MIR156c* could have epistatic interactions with more than one SNP in the three potential targets of *Pto-miR156c* (Figure 4B). For example, SNP9 from *Pto-MIR156c* formed 12 SNP pairs with SNPs from the three candidate targets, with pairwise effects of 0.33–5.81% on phenotypic traits.

**Table 4:** **Summary of all significant SNP pairs associated with each trait under epistasis model in the association population of ***P. tomentosa*****.

**Traits**	**Number of associations**	**Number of SNPs**				**Range of interaction effect (%)**	**Range of IGs (%)**
		***Pto-MIR156c***	***Pto-SPL15***	***Pto-SPL20***	***Pto-SPL25***		
DBH (cm)	5	1	–	2	1	1.14–8.41	−1.24 to 3.26
H (m)	9	–	3	3	–	0.28–4.03	−3.82 to 1.67
V (m^3^)	9	–	3	3	–	0.11–3.48	−2.47 to 1.05
FL(mm)	3	1	1	1	–	3.48–3.50	−4.29 to 0.46
FW (μm)	13	2	2	1	1	0.08–8.70	−8.66 to 1.16
MFA (°)	11	–	3	1	2	0–2.38	−7.47 to −0.56
HC (%)	9	–	1	4	1	0.31–3.82	−3.09 to 2.46
CC (%)	9	1	4	1	–	0.01–4.84	−4.39 to 1.37
HEC (%)	13	1	1	2	2	1.13–5.81	−5.23 to −0.07
LC (%)	11	1	3	2	–	0–2.50	−4.23 to 0.82

To decipher the pairwise effect for tree growth and wood property traits, we constructed three interaction graphs for FW and HEC, and found that each graph revealed two-way interactions between four SNPs in *Pto-MIR156c, Pto-SPL15, Pto-SPL20*, and *Pto-SPL25* (Figure 4B). The nine SNPs had single effects for the associated traits of 0–7.45%. Among the 15 interactions, 13 had negative IGs, indicating redundant information in these SNP pairs. Interestingly, *Pto-MIR156c*-SNP10 had no effect on FW, while it did have pairwise effects on FW when in combination with *Pto-SPL15*-SNP75, *Pto-SPL20*-SNP47, and *Pto-SPL25*-SNP55 (Figure 4B). In addition, the SNP pair of *Pto-MIR156c*-SNP15 and *Pto-SPL20*-SNP5 had epistatic interactions on CC, and the higher and lower values of different genotypic combinations from two SNPs significantly differed from the values of the single-locus genotype (Figures 4C–G). For example, epistasis values of the CG-CT combination (0.563%) in the SNP pair *Pto-MIR156c*-SNP15 and *Pto-SPL20*-SNP5 were significantly higher than any of the single-genotype values (0.063% for CG and 0.167% for CT). However, the genotypic combinations of *Pto-MIR156c*-SNP15 and *Pto-SPL15*-SNP58 displayed a different situation, in which only CG-AG genotype combination produced epistasis values that differed from the single-locus genotype. All these findings can be used as evidence for dissecting gene-gene epistatic interactions.

### Transcript analysis of significant SNP genotypes

**Figure 5:**
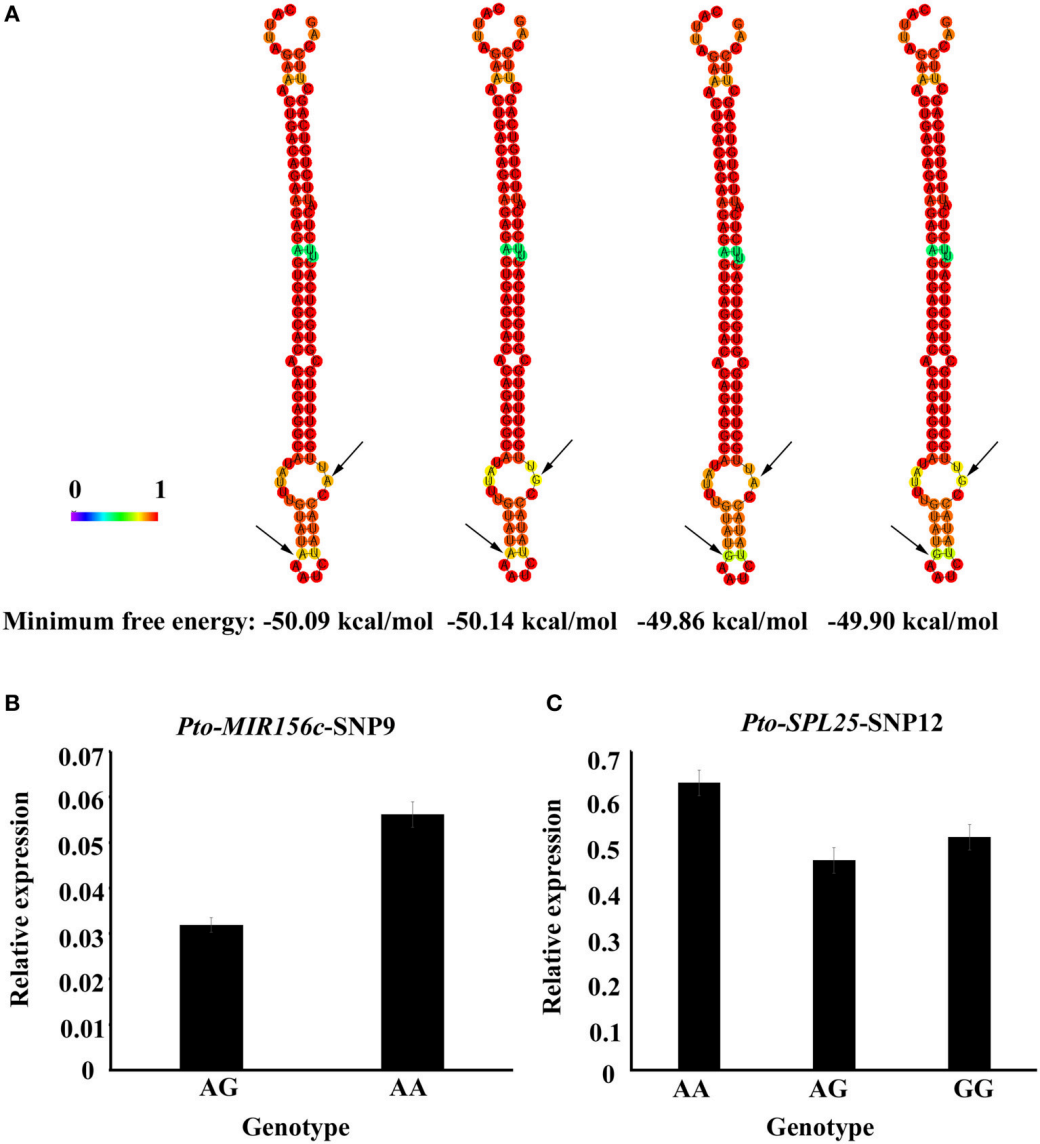
**Secondary structures with SNPs in the pre-miRNA region of ***Pto-MIR156c*** and transcript abundance of SNP genotypes for ***Pto-MIR156c*** and ***Pto-SPL25***. (A)** The secondary structures of *Pto-MIR156c* pre-miRNA sequence affected by two SNPs in the pre-miRNA region, and the minimum free energy of the four secondary structures. **(B,C)** Transcript levels (arbitrary units normalized to control) of *Pto-MIR156c* **(B)** and *Pto-SPL25* **(C)** in different genotypic classes for *Pto-MIR156c-SNP9* and *Pto-SPL25-SNP12*, respectively.

To further investigate the roles of SNPs in pre-miRNA region of *Pto-MIR156c*, we predicted four secondary structures by RNAfold, based on the two SNPs in the pre-miRNA region of *Pto-MIR156c* (*Pto-MIR156c*-SNP9 and *Pto-MIR156c*-SNP10) (Figure 5A). The results showed that the two SNPs slightly altered the minimum free energy (from −50.14 kcal/mol to −49.86 kcal/mol) of the predicted secondary structure of *Pto-MIR156c*. Then, we used RT-qPCR to measure the transcript abundance of *Pto-MIR156c* and the effect of SNPs within the pre-miRNA region. The results showed that *Pto-MIR156c*-SNP9 produced significant differences in *Pto-MIR156c* transcript abundance in the different genotypes, with the highest expression levels in the AA group (0.056 ± 0.003, arbitrary units normalized to control), and the lowest levels in the AG group (0.032 ± 0.002) (Figure 5B). We also tested the transcript abundance of target genes affected by significant SNPs. For example, three genotypes of *Pto-SPL25*-SNP12 caused different expression levels of *Pto-SPL25* (Figure 5C), with the highest levels in the AA group (0.63 ± 0.03), followed by the GG (0.51 ± 0.02) group, and the lowest in the AG group (0.46 ± 0.02).

## Discussion

### Characterization of *Pto-MIR156c* and its complex regulatory network with *Pto-SPLs* in *P. tomentosa*

In perennial trees, most biological processes, such as tree growth and wood formation, are regulated by coordinated and elaborate networks, including non-coding RNA (ncRNA)-mRNA, mRNA-mRNA, and ncRNA-ncRNA interactions (Lu et al., 2013). Of these, miRNAs, a class of crucial ncRNAs, function as key regulators of gene expression. Moreover, SNPs in miRNA genes may act as functional markers by affecting the pre-miRNA secondary structure, altering miRNA abundance, and modifying the expression of the targets, thus affecting phenotypic variation (Ryan et al., 2010). For example, three SNPs in the pre-miRNA region of *Pto-MIR397a* strongly affected the stability of the secondary structure, and associate with tree growth and wood properties in *P. tomentosa* (Chen et al., 2015). Here, we identified two SNPs (*Pto-MIR156c*-SNP9 and *Pto-MIR156c*-SNP10) in the pre-miRNA region of *Pto-MIR156c*, that lead to slight changes of the minimum free energy of its secondary structure (Figure 5A), which could affect the stability of this secondary structure. Furthermore, *Pto-MIR156c*-SNP9 affected the transcript abundance of *Pto-MIR156c* (Figure 5B). The altered stability of the secondary structure could affect the transcript accumulation of *Pto-MIR156c*; beyond that, the two SNPs could also alter transcription factor binding sites or processing signals, which might affect the transcription of the miRNA. This needs to be analyzed in future studies. In addition, the significant positive expression correlation of *Pto-MIR156c* gene and mature *miR156* (Figure S5) revealed that the SNPs in pre-miRNA region may affect the abundance of mature miRNA. In addition to this, the processing efficiency could also be considered as a factor in affecting the maturation of miRNA.

In addition, single SNP-based and haplotype-based association analysis in the natural population found that *Pto-MIR156c*-SNP9 and *Pto-MIR156c*-SNP10 significantly associated with CC and HEC. Taken together, our findings support the idea that SNPs in the pre-miRNA could affect the transcript abundance of the miRNA gene and contribute to phenotypic variations. In the flanking region of *Pto-MIR156c*, 15 common SNPs (MAF > 0.05) were found with nucleotide diversity of π = 0.01159, which was higher than that found in the pre-miRNA region of *Pto-MIR156c* (π = 0.01023), and no SNPs were detected in the mature region. Alignment results showed the mature sequences of *miR156c* in *P. tomentosa, P. trichocarpa, O. sativa, Z. mays*, and *A. thaliana* were completely conserved, which strongly emphasized that the mature miRNAs are highly conserved (Reinhart et al., 2002). Also, expression analysis failed to detect the expression of *Pto-MIR156c* in the shoot apex, and the expression levels of other tissues showed a varied pattern, revealing that *Pto-MIR156c* is expressed in a tissue-specific manner (Puzey et al., 2012). We also found that *Pto-MIR156c* expression was higher in cambium, indicating that it may have potential regulatory roles in primary cell wall formation. In addition, *MIR156h* also exhibited high expression levels in xylem (Kozomara and Griffiths-Jones, 2014; Figure S1), suggesting that *miR156h* might be essential in regulating tree growth and wood formation. However, *MIR156h* is conserved in our association population of *P. tomentosa*, which makes it inappropriate to decipher the vital roles of *miR156h* in tree development by association studies. We can analyze the regulatory roles of *miR156h* through other strategies in future.

This study identified *Pto-SPL15, Pto-SPL20*, and *Pto-SPL25* as potential targets of *Pto-miR156c* through psRNATarget prediction and degradome sequencing (Figure 1B). Expression pattern analysis also showed that *Pto-MIR156c* and its three potential targets might participate in wood formation through a shared pathway (Figure 1C). Previous studies found that miRNAs negatively regulate the expression of their targets, while a few distinctive findings were detected in our studies. For example, the expression of *Pto-MIR156c* and *Pto-SPL15, Pto-SPL20*, and *Pto-SPL25* exhibited weak or no correlation (*r* = 0.17, −0.04, and −0.42, respectively, *P* < 0.05) in seven tested tissues and organs. Also, *Pto-SPL20* and *Pto-SPL25* showed strong negative correlations with *Pto-miR156c* in stem tissues (phloem, cambium, developing xylem, and mature xylem), suggesting that the regulation of *Pto-SPL20* and *Pto-SPL25* by *Pto-miR156c* occurred in a tissue-specific manner.

By contrast, *Pto-miR156c* and *Pto-SPL15* showed a positive correlation (*r* = 0.40, *P* < 0.05) in stem, inconsistent with former studies. Several reasons might explain this phenomenon. For example, the *Pto-SPL15* gene might be regulated by more than one miRNA, or other members in the same miRNA family or other miRNA families might contribute more to the regulation of *Pto-SPL15* expression (Jens and Rajewsky, 2014). It is the possibility that *Pto-miR156c* might regulate *Pto-SPL15* mainly via translational suppression; therefore, we cannot see a clear expression reduction of *Pto-SPL15* in transcriptional level. There is also a possibility that the transcript levels of *Pto-SPL15* was mainly affected by its own transcriptional regulation. Due to the specificity of miRNA expression in different developmental stages and tissues, it is probable that the negative regulation of *Pto-miR156c* and *Pto-SPL15* might not be reflected in the tested tissues and developmental stages. For example, RNA-binding proteins, such as Argonaute and Dicer, regulate the accessibility of individual sites in a tissue- and stage-specific manner, which could affect the regulation of miRNA and mRNA (Hausser and Zavolan, 2014). Another possibility is that feedback mechanisms could counteract the effect of the miRNA-mRNA interaction (Hausser and Zavolan, 2014). For example, in humans, Dicer is a key enzyme in miRNA biogenesis and is controlled by the miRNA let-7. Knockdown of let-7 strongly deregulated the production of Dicer, which in turn increased the abundance of other miRNAs, and thus indirectly reduced the expression of the corresponding mRNAs (Selbach et al., 2008). In addition, competing ncRNAs, such as long non-coding RNAs (lncRNAs), could affect this phenomenon, possibly by specifically competing for miRNA binding (Hausser and Zavolan, 2014). For example, in animals, lncRNA H19 acts as a sponge for miRNA let-7, thereby increasing the expression of let-7-regulated genes, promoting muscle differentiation, and the miRNA-mRNA regulatory system was also regulated by the relative concentrations of miRNA, lncRNA, and mRNA (Kallen et al., 2013). Taken together our results indicate that, except for the direct regulation of *Pto-miR156c* and *Pto-SPL15*, there might be an alternative pathway, in which *Pto-miR156c* could indirectly regulate the expression of *Pto-SPL15*. We can examine this in future studies.

### SNPs in *Pto-MIR156c* and the three potential targets of *Pto-miR156c* associated with tree growth and wood property traits

Our study detected 69 and 72 significant associations (*P* < 0.01) via single-SNP-based and haplotype-based association studies, respectively, which illustrated that *Pto-miR156c* and its three putative targets might function in wood formation through the same pathway (Figure 3A). For *Pto-MIR156c*, the haplotype of A-A and G-G in *Pto-MIR156c*-SNP9-10 associated with HEC, which was supported by single-SNP-based association (Figure 3B), and the haplotype A-A also associated with CC, revealing the pleiotropy of this gene and its roles in wood formation. In addition, one SNP from the flanking region of *Pto-MIR156c* associated with HC, and five haplotypes from SNPs in the flanking region associated with three traits (DBH, FW, and HEC), suggesting that SNPs in flanking regions could also cause phenotypic variation, consistent with the idea that SNPs within flanking regions function in regulating the roles of miRNAs (Zeng and Cullen, 2005). These association studies in *Pto-MIR156c* illustrate the crucial roles of *Pto-miR156c* in tree growth and wood formation, and provide evidence for dissecting the functional SNPs in *Pto-MIR156c*.

Also, 48 common SNPs (MAF > 0.05) in *Pto-SPL15, Pto-SPL20*, and *Pto-SPL25* associated with six traits related to tree growth and wood properties with different *R*^2^, indicating the possible roles of *SPL* genes in tree growth and wood properties. Of the associated SNPs, six were in exons, which could affect phenotypic variation by causing non-synonymous mutations or influencing expression levels (Kimchi-Sarfaty et al., 2007). For example, three genotypes of *Pto-SPL20*-SNP12 influenced the expression level of *Pto-SPL20* (Figure 5C). Also, *Pto-SPL20*-SNP21, caused a missense mutation of Ala to Gly, associated with V.

In general, 86.96% of the associations showed additive effects, and 57.97% displayed dominant effects, which indicated that additive effects may play relatively major roles in genetic variation (Figure 3A). Both additive and dominant effects offered clues to the common roles of *Pto-miR156c* and its three putative targets on tree growth and wood formation, and provided significant resources for applications in marker-assisted breeding of trees.

Furthermore, at least one SNP of three target genes associated with the same trait as *Pto-MIR156c*. For instance, *Pto-MIR156c*-SNP9, *Pto-MIR156c*-SNP10, *Pto-SPL15*-SNP106, and *Pto-SPL20*-SNP8 simultaneously associated with HEC with different additive and dominant effects and *R*^2^. Many haplotypes in the four candidate genes associated with one trait. For example, five haplotypes from *Pto-MIR156c*, seven haplotypes from *Pto-SPL15*, nine haplotypes from *Pto-SPL20*, and three haplotypes from *Pto-SPL25* simultaneously associated with FW, explaining 0.68–21.73% of the phenotypic variance. These findings indicate that *Pto-miR156c* and its three putative targets may co-regulate the phenotypic variation by sharing the same regulatory pathway. In addition, one SNP or one haplotype associated with more than one trait with different contributions; for example, *Pto-SPL25*-SNP30 associated with FW and DBH with *R*^2^ of 13.33 and 13.50%, respectively, revealing the functional roles of the locus and pleiotropy of the gene.

In summary, the association studies, combined with the correlation of expression patterns in stem, illustrate the common roles of *miR156c* and its three potential targets in tree growth and wood formation, and enlarge our understanding of the potential roles of the miR156c-*SPL* regulatory system in wood formation in perennial woody plants.

### The interaction of *Pto-miR156c* and its potential targets on wood formation revealed by epistasis effects

Epistasis has been regarded as an essential effect for understanding molecular evolution and a feasible tool for identifying the effects of genetic variation and interactions among multiple genes on quantitative traits in breeding (Mackay, 2014). Also, epistasis provides necessary and complementary information to that gained from single-locus analysis, and few studies have used epistasis analysis to decipher the regulatory networks of multiple genes in trees (Du et al., 2015). Here, we identified 129 significant pairwise associations, and 85.27% of them showed negative IG, indicating the redundant information carried in the two SNPs and their similar roles in the same traits (Figure 4A). The associations with positive IGs indicate that pairwise interactions provide greater contributions to phenotypic variation than the sum of the individual contributions of the two SNPs. Both the positive and negative IGs illustrate the close genetic interactions of the miRNA and its targets.

We found that 21.71% of the SNP pairs were miRNA-mRNA interactions, reflecting the possible genetic interactions of *Pto-miR156c* and its putative targets on tree growth and wood formation, while 49.61% of the SNP-SNP interactions were from two target genes, revealing the interactions of target genes could also contribute to wood formation.

Interestingly, one SNP in *Pto-MIR156c* could form multiple SNP pairs with SNPs in the three putative targets. For example, *Pto-MIR156c*-SNP9 showed epistasis interactions with *Pto-SPL15*-SNP89, *Pto-SPL20*-SNP20, and *Pto-SPL25*-SNP51 (Figure 4B). The six SNP pairs acted on HEC with pairwise effects of 0.08 to 8.70%, suggesting the complexity of the interaction between *Pto-miR156c* and its three potential targets for wood formation and the predominant roles of *Pto-MIR156c* in the regulatory network for phenotypic variation.

In the epistasis model, some loci possessed significant roles only when they interacted with other SNPs, such as *Pto-SPL20*-SNP1 and *Pto-MIR156c*-SNP10, illustrating the significance of the complicated regulatory network. In addition, epistatic effects of different genotype combinations could also be treated as evidence of the effects of gene interactions on quantitative traits (Figures 4C–G). For example, the epistasis effect of CG-CT combinations (0.563%) from *Pto-MIR156c*-SNP15 and *Pto-SPL20*-SNP5 for CC was significantly higher than any of the single genotypes (0.063% for CG and 0.167% for CT) (Figures 4C,D,F), indicating that the genotype combinations had greater phenotypic contributions than single loci. By contrast, the effect of the AG genotype on CC in *Pto-MIR156c*-SNP15 was masked when the genotype of *Pto-SPL15*-SNP58 was AA and GG (Figures 4E,G), which indicated the epistasis of the SNP pairs and their interactions for wood formation. However, the detailed regulatory mechanisms and the functional SNPs identified under epistasis models also need to be examined in future studies.

## Conclusions

Here, we conducted association studies and expression profiling to examine the roles and interactions of *Pto-miR156c* and its potential targets in tree growth and wood formation. Single SNP-based association studies with underlying additive and dominant effects and haplotype-based association studies, combined with expression pattern analysis, provided genetic evidence of the potential common roles and correlation of *Pto-miR156c* and its three putative targets in wood formation. Epistasis analysis, which opened a feasible pathway to uncover genetic variation and define the genetic regulatory networks of miRNA-mRNA interactions in the population genetics of trees, deciphered the epistasis interactions of *Pto-MIR156c* and the three potential targets and dissected their potential roles in wood formation. In addition, this study revealed the potential roles of the *miR156-SPL* system in wood formation in trees. Overall, our studies contributed to studying the genetic variations and functions of miRNA-mRNA interactions, and the functional SNPs in our studies could be used in future studies.

## Author contributions

DZ designed the conception and experiment; MQ and QW performed the experiments; SP helped to collect and analyze the data; MQ wrote the manuscript; QW and SP provided valuable suggestions on the manuscript; XY, YS, QD, and DZ revised the manuscript; DZ obtained funding and is responsible for this article. All authors read and approved the manuscript.

## Data archiving statement

Sequence data in this article have been deposited with the GenBank Data Library under the accession numbers KX079977 –KX080148, and the degradome sequencing data are available in SRA database under the accession number SRX1447192.

### Conflict of interest statement

The authors declare that the research was conducted in the absence of any commercial or financial relationships that could be construed as a potential conflict of interest.

## Supplementary material

Figure S1 **The expression patterns of ***Pto-MIR156*** gene family in different tissues and organs**.

Figure S2 **The most likely cleavage sites between ***Pto-miR156c*** and ***Pto-SPL15*** identified by degradome sequencing**.

Figure S3 **The most likely cleavage sites between ***Pto-miR156c*** and ***Pto-SPL20*** identified by degradome sequencing**.

Figure S4 **The most likely cleavage sites between ***Pto-miR156c*** and ***Pto-SPL25*** identified by degradome sequencing**.

Figure S5 **The expression correlation analysis of ***Pto-MIR156c*** and mature ***miR156*****.

Table S1 **The primers used for real-time PCR in this study**.

Table S2 **Single-nucleotide polymorphisms of ***Pto-MIR156c*** and the three potential targets of ***Pto-miR156c*****.

Table S3 **SNPs in ***Pto-MIR156c, Pto-SPL15, Pto-SPL20***, and ***Pto-SPL25*** identified in our studies**.

Table S4 **Details of significant SNPs within ***Pto-MIR156c*** and the three potential targets of ***Pto-miR156c*** associated with growth and wood properties in an association population of ***P. tomentosa*****.

Table S5 **Significant haplotypes from ***Pto-MIR156c*** and its potential targets associated with growth and wood properties in the association population**.

Table S6 **The SNP pairs and their main effects detected from Pto-MIR156c and the three potential targets of ***Pto-miR156c*** under an epistasis model in the association population of ***P. tomentosa*****.
