# Supplementary figures and images for: Association Studies in Populus tomentosa Reveal the Genetic Interactions of Pto-MIR156c and Its Targets in Wood Formation
Source: Front Plant Sci. 2016 Aug 3;7:1159. doi: 10.3389/fpls.2016.01159 (PMC4971429; doi:10.3389/fpls.2016.01159)

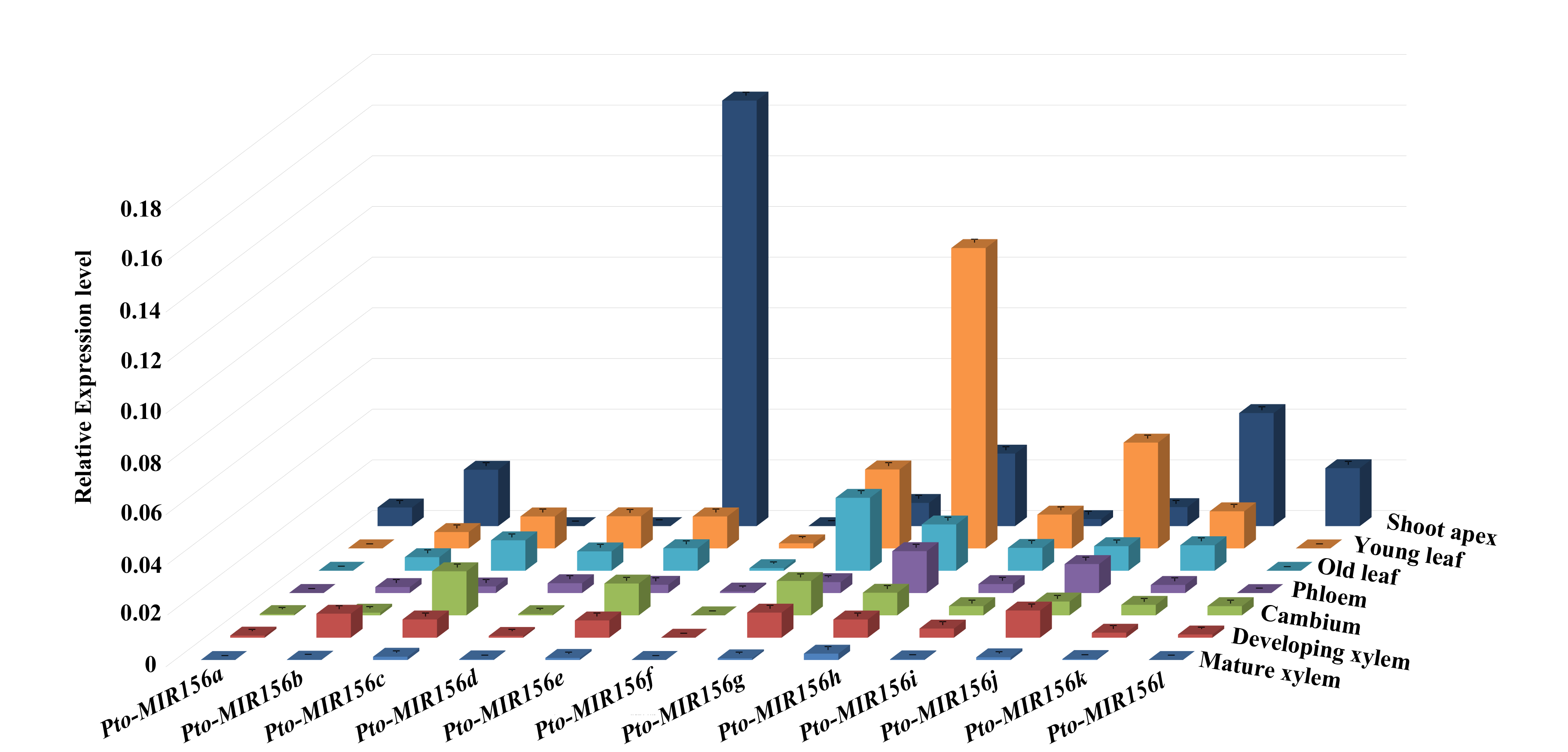

Supplement: Figure S1 — The expression patterns of Pto-MIR156 gene family in different tissues and organs. [file Image1.TIF]

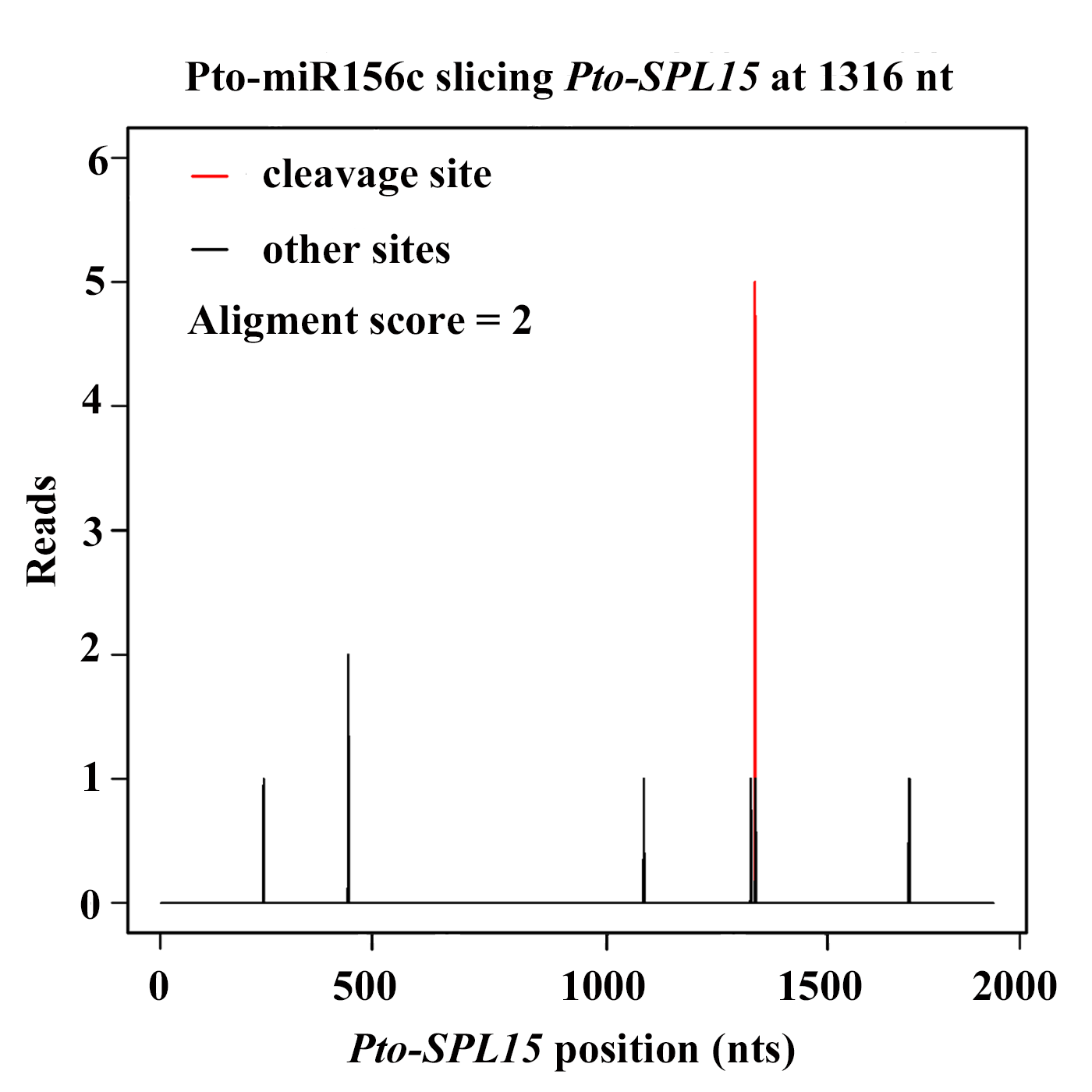

Supplement: Figure S2 — The most likely cleavage sites between Pto-miR156c and Pto-SPL15 identified by degradome sequencing. [file Image2.TIF]

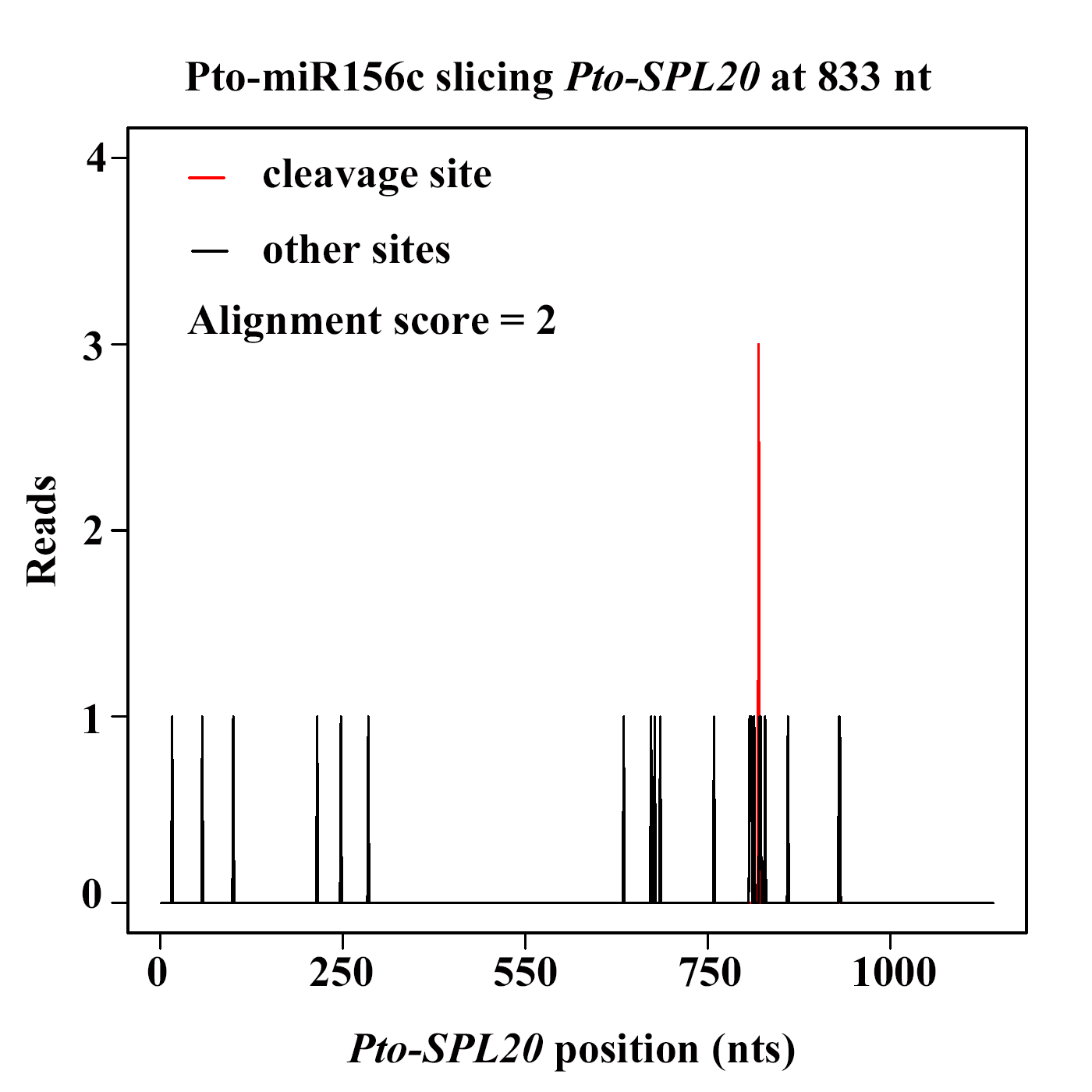

Supplement: Figure S3 — The most likely cleavage sites between Pto-miR156c and Pto-SPL20 identified by degradome sequencing. [file Image3.TIF]

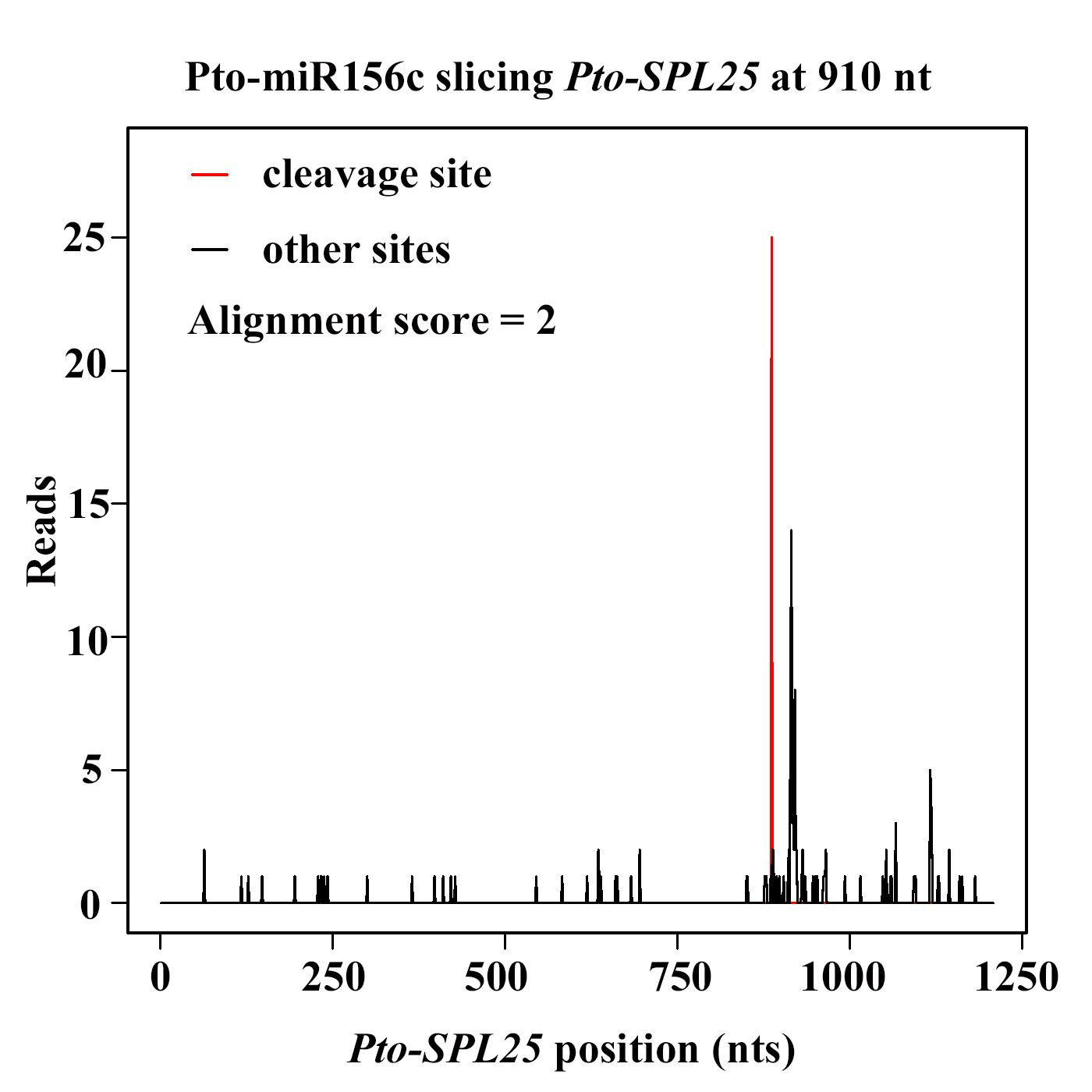

Supplement: Figure S4 — The most likely cleavage sites between Pto-miR156c and Pto-SPL25 identified by degradome sequencing. [file Image4.TIF]

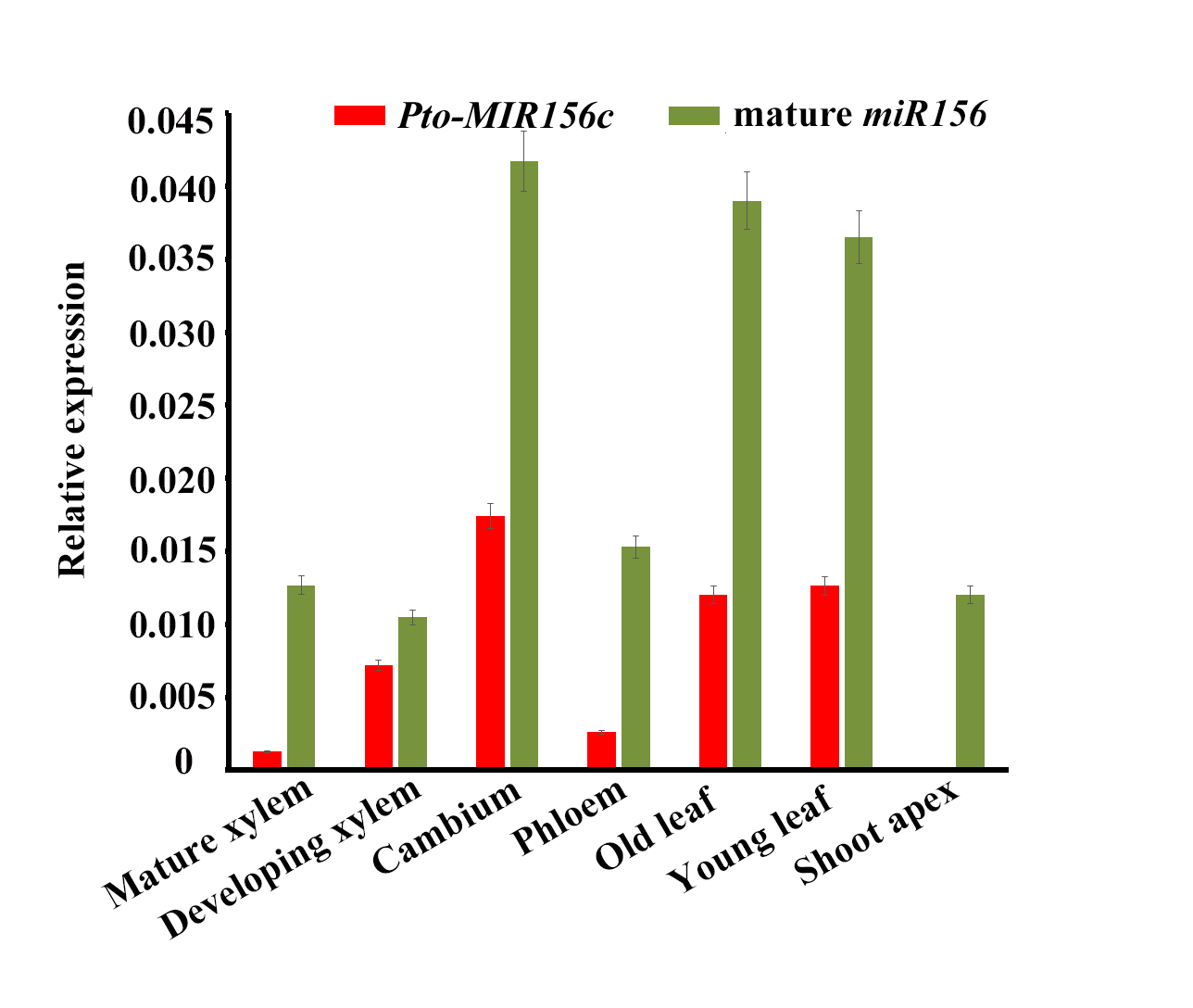

Supplement: Figure S5 — The expression correlation analysis of Pto-MIR156c and mature miR156. [file Image5.tif]
