# Supplementary material for: Association Studies in Populus tomentosa Reveal the Genetic Interactions of Pto-MIR156c and Its Targets in Wood Formation
Source: Front Plant Sci. 2016 Aug 3;7:1159. doi: 10.3389/fpls.2016.01159 (PMC4971429; doi:10.3389/fpls.2016.01159)
Supplement: Table S1 — The primers used for real-time PCR in this study. [file Table1.DOC]

| **Gene** | **Forward primer(5′→3′ )** | **Reverse primer (5′→3′ )** |
| --- | --- | --- |
| *Pto-MIR156a* | CTGACAGAAGAGAGTGAGCACACA | GTGAGCACACACCCTGCAAT |
| *Pto-MIR156b* | AGGGAGGTGACAGAAGAGAGTGA | AAAGTTTCAAGCATGGATGTCATG |
| *Pto-MIR156c* | TGAGCACACAGAGGCATATTTGT | GGAAGCTGACAGAATGAGAAGTGA |
| *Pto-MIR156d* | TTGACAGAAGAGAGTGAGCACACA | GCACGCAAAGCTTCAAGCA |
| *Pto-MIR156e* | ATCCTCCACCCAGAGCTCAA | AACGGGAAAGAAAACAAGCAAGT |
| *Pto-MIR156f* | CTGACAGAAGAGAGTGAGCACACA | AGCACACACCCTGCAATGG |
| *Pto-MIR156g* | CACTGATGATGAAATGCATGGA | GCTGAAGGTGATGACAGGAGACT |
| *Pto-MIR156h* | GCATGGAGCTTGATTGCATCT | GGCTGAAGGTGATGACAGAAGAC |
| *Pto-MIR156i* | GAGCACAGATGATGTTTTGCAGTA | GGCAGAAGCATAGAGAGCACAA |
| *Pto-MIR156j* | GAAGGAGGCACTGTTGATGTTG | GCAGAGTCCATTGCATATCATCAT |
| *Pto-MIR156k* | GGGAGCACAACCCTGTAATAGC | TTGGTGACAGAAGAAGAGAAAGCA |
| *Pto-MIR156l* | GACAGAAGATGGAGAGCACAGCTA | AAACACAAAGCACAAACTGCACTT |
| *Pto-SPL15* | ATCTGCATCAACTTCCTGACAAAC | CCCCCTTTGTAGCTGTTCGA |
| *Pto-SPL20* | TCCCACCCCAAGGAAGTTC | GCTGGTTTGGTCTTCATCTGATC |
| *Pto-SPL25* | GCGTTTGGCAGGACACAAT | TCTCCATAAGATTCAGCCGTGTT |
| *Actin* | TTCATTTCACATCTTCCCCTTTT | GATCTCTGTGTGGGCGTCTGT |
| *Pto-miR156* | TGACAGAAGAGAGTGAGCAC | GTCGTATTAATTCTGTGCTCGC |
| 5.8S rRNA | GTCTGCCTGGGTGTCACGCAA | GTCGTATTAATTCTGTGCTCGC |
| Poly(T) adapter | GCGAGCACAGAATTAATACGACTCACTATAGG(T)12VN* | |

Table S1 The primers used for real-time PCR in this study.

*V = A, G, C; N = A, T, G, C
