# Supplementary material for: Association Studies in Populus tomentosa Reveal the Genetic Interactions of Pto-MIR156c and Its Targets in Wood Formation
Source: Front Plant Sci. 2016 Aug 3;7:1159. doi: 10.3389/fpls.2016.01159 (PMC4971429; doi:10.3389/fpls.2016.01159)
Supplement: Table S2 — Single-nucleotide polymorphisms of Pto-MIR156c and the three potential targets of Pto-miR156c. [file Table2.DOC]

**Table S2** Single-nucleotide polymorphisms of *Pto-MIR156c* and the three potential targets of *Pto-miR156c*.

| **Gene** | **Region** | **Length**  **(bp)** | **Number of polymorphic sites** | **Number of common SNPs** | **Percentage polymorphism (%)** | **Nucleotide diversity** | | |
| --- | --- | --- | --- | --- | --- | --- | --- | --- |
|  |  |  |  |  |  | **π** | **θw** | |
| *Pto-MIR156c* |  |  |  |  |  |  |  |  |
|  | Flanking region | 600 | 22 | 15 | 3.67 | 0.0116 | 0.0085 |  |
|  | Pre-mature region | 100 | 2 | 2 | 2.00 | 0.0102 | 0.0046 |  |
|  | Mature region | 20 | 0 | 0 | 0.00 | 0 | 0 |  |
|  | Total | 700 | 24 | 17 | 3.43 | 0.0114 | 0.0079 |  |
| *Pto-SPL15* |  |  |  |  |  |  |  |  |
|  | Promoter | 2000 | 62 | 48 | 3.10 | 0.0082 | 0.0072 |  |
|  | 5'UTR | 964 | 17 | 6 | 1.76 | 0.0026 | 0.0041 |  |
|  | Intron in 5'UTR | 555 | 11 | 4 | 1.98 | 0.0029 | 0.0046 |  |
|  | Exon1 | 419 | 21 | 20 | 5.01 | 0.0139 | 0.0116 |  |
|  | Intron1 | 619 | 21 | 8 | 3.39 | 0.0058 | 0.0078 |  |
|  | Exon2 | 134 | 1 | 0 | 0.75 | 0.0007 | 0.0017 |  |
|  | Intron2 | 558 | 16 | 3 | 2.87 | 0.0035 | 0.0066 |  |
|  | Exon3 | 434 | 16 | 15 | 3.69 | 0.0073 | 0.0085 |  |
|  | 3'UTR | 454 | 11 | 10 | 2.42 | 0.0073 | 0.0056 |  |
|  | Flanking region | 500 | 1 | 1 | 0.20 | 0.0005 | 0.0005 |  |
|  | Synonymous | 221.11 | 9 | 8 | 4.07 | 0.0094 | 0.0094 |  |
|  | Nonsynonymous | 762.89 | 29 | 27 | 3.80 | 0.0092 | 0.0088 |  |
|  | Total silent^a^ | 5319.1 | 137 | 84 | 2.58 | 0.0057 | 0.006 |  |
|  | Total^b^ | 6082 | 166 | 111 | 2.73 | 0.0061 | 0.0064 |  |
| *Pto-SPL20* |  |  |  |  |  |  |  |  |
|  | Promoter | 1784 | 60 | 38 | 3.36 | 0.0067 | 0.0078 |  |
|  | 5'UTR | 216 | 4 | 2 | 1.85 | 0.0039 | 0.0043 |  |
|  | Exon1 | 353 | 8 | 4 | 2.27 | 0.0033 | 0.0052 |  |
|  | Intron | 991 | 37 | 23 | 3.73 | 0.0073 | 0.0086 |  |
|  | Exon2 | 238 | 4 | 2 | 1.68 | 0.0017 | 0.0039 |  |
|  | 3'UTR | 254 | 11 | 6 | 4.33 | 0.0058 | 0.01 |  |
|  | Flanking region | 500 | 14 | 14 | 2.80 | 0.0082 | 0.0065 |  |
|  | Synonymous | 127.88 | 4 | 2 | 3.13 | 0.0028 | 0.0072 |  |
|  | Nonsynonymous | 460.12 | 8 | 4 | 1.74 | 0.0026 | 0.004 |  |
|  | Total silent^a^ | 3875.9 | 130 | 85 | 3.35 | 0.0067 | 0.0078 |  |
|  | Total^b^ | 4336 | 138 | 89 | 3.18 | 0.0062 | 0.0074 |  |
| *Pto-SPL25* |  |  |  |  |  |  |  |  |
|  | Promoter | 2000 | 23 | 23 | 1.15 | 0.0038 | 0.0027 |  |
|  | 5'UTR | 156 | 4 | 4 | 2.56 | 0.011 | 0.0059 |  |
|  | Exon1 | 491 | 7 | 7 | 1.43 | 0.005 | 0.0033 |  |
|  | Intron | 1010 | 21 | 21 | 2.08 | 0.0085 | 0.0048 |  |
|  | Exon2 | 238 | 4 | 4 | 1.68 | 0.0053 | 0.0039 |  |
|  | 3'UTR | 274 | 3 | 3 | 1.09 | 0.0037 | 0.0025 |  |
|  | Flanking region | 500 | 4 | 4 | 0.80 | 0.002 | 0.0019 |  |
|  | Synonymous | 163.06 | 4 | 4 | 2.45 | 0.0102 | 0.0057 |  |
|  | Nonsynonymous | 562.94 | 7 | 7 | 1.24 | 0.0036 | 0.0029 |  |
|  | Total silent^a^ | 4106.1 | 59 | 59 | 1.44 | 0.0052 | 0.0033 |  |
|  | Total^b^ | 4669 | 66 | 66 | 1.41 | 0.005 | 0.0033 |  |

^a^Total silent: synonymous sites plus polymorphic sites in noncoding regions of genes*.*

^b^Total: silent sites plus nonsynonymous sites of genes.
