# Supplementary material for: Association Studies in Populus tomentosa Reveal the Genetic Interactions of Pto-MIR156c and Its Targets in Wood Formation
Source: Front Plant Sci. 2016 Aug 3;7:1159. doi: 10.3389/fpls.2016.01159 (PMC4971429; doi:10.3389/fpls.2016.01159)
Supplement: Table S3 — SNPs in Pto-MIR156c, Pto-SPL15, Pto-SPL20, and Pto-SPL25 identified in our studies. [file Table3.DOC]

**Table S3** SNPs in *Pto-MIR156c*, *Pto-SPL15*, *Pto-SPL20*, and *Pto-SPL25* identified in our studies.

| **Gene** | **SNP locus** | **SNP position^a^** | **Genotype** | **Region** |
| --- | --- | --- | --- | --- |
| *Pto-MIR156c* |  |  |  |  |
|  | SNP1 | 13 | T>A | Flanking region |
|  | SNP2 | 34 | T>G | Flanking region |
|  | SNP3 | 56 | A>C | Flanking region |
|  | SNP4 | 216 | T>C | Flanking region |
|  | SNP5 | 267 | G>A | Flanking region |
|  | SNP6 | 268 | G>A | Flanking region |
|  | SNP7 | 283 | G>T | Flanking region |
|  | SNP8 | 286 | T>C | Flanking region |
|  | SNP9 | 349 | A>G | Pre-miRNA region |
|  | SNP10 | 360 | A>G | Pre-miRNA region |
|  | SNP11 | 452 | T>C | Flanking region |
|  | SNP12 | 527 | G>A | Flanking region |
|  | SNP13 | 549 | C>T | Flanking region |
|  | SNP14 | 574 | T>G | Flanking region |
|  | SNP15 | 582 | G>C | Flanking region |
|  | SNP16 | 669 | T>C | Flanking region |
|  | SNP17 | 677 | A>T | Flanking region |
| *Pto-SPL15* |  |  |  |  |
|  | SNP1 | 5604 | T>A | Flanking^b^ |
|  | SNP2 | 5500 | A>G | 3'UTR |
|  | SNP3 | 5488 | T>A | 3'UTR |
|  | SNP4 | 5484 | T>C | 3'UTR |
|  | SNP5 | 5483 | G>A | 3'UTR |
|  | SNP6 | 5475 | A>T | 3'UTR |
|  | SNP7 | 5362 | G>A | 3'UTR |
|  | SNP8 | 5396 | G>A | 3'UTR |
|  | SNP9 | 5267 | T>G | 3'UTR |
|  | SNP10 | 5248 | A>G | 3'UTR |
|  | SNP11 | 5157 | G>T | 3'UTR |
|  | SNP12 | 5000 | A>T | Exon3 |
|  | SNP13 | 4979 | A>T | Exon3 |
|  | SNP14 | 4956 | G>A | Exon3 |
|  | SNP15 | 4843 | G>A | Exon3 |
|  | SNP16 | 4791 | G>A | Exon3 |
|  | SNP17 | 4773 | C>G | Exon3 |
|  | SNP18 | 4770 | C>T | Exon3 |
|  | SNP19 | 4764 | C>A | Exon3 |
|  | SNP20 | 4753 | T>G | Exon3 |
|  | SNP21 | 4746 | T>C | Exon3 |
|  | SNP22 | 4742 | A>G | Exon3 |
|  | SNP23 | 4719 | G>A | Exon3 |
|  | SNP24 | 4712 | T>C | Exon3 |
|  | SNP25 | 4703 | G>T | Exon3 |
|  | SNP26 | 4698 | C>A | Exon3 |
|  | SNP27 | 4647 | C>A | Intron2 |
|  | SNP28 | 4629 | A>G | Intron2 |
|  | SNP29 | 4378 | A>G | Intron2 |
|  | SNP30 | 3967 | C>T | Intron1 |
|  | SNP31 | 3761 | C>T | Intron1 |
|  | SNP32 | 3692 | A>G | Intron1 |
|  | SNP33 | 3682 | A>G | Intron1 |
|  | SNP34 | 3585 | T>C | Intron1 |
|  | SNP35 | 3431 | C>T | Intron1 |
|  | SNP36 | 3429 | T>C | Intron1 |
|  | SNP37 | 3390 | A>C | Intron1 |
|  | SNP38 | 3340 | A>G | Exon1 |
|  | SNP39 | 3290 | A>G | Exon1 |
|  | SNP40 | 3283 | A>G | Exon1 |
|  | SNP41 | 3265 | T>A | Exon1 |
|  | SNP42 | 3263 | A>G | Exon1 |
|  | SNP43 | 3219 | T>A | Exon1 |
|  | SNP44 | 3199 | A>G | Exon1 |
|  | SNP45 | 3198 | A>C | Exon1 |
|  | SNP46 | 3186 | G>A | Exon1 |
|  | SNP47 | 3180 | G>A | Exon1 |
|  | SNP48 | 3178 | G>C | Exon1 |
|  | SNP49 | 3101 | A>T | Exon1 |
|  | SNP50 | 3097 | A>G | Exon1 |
|  | SNP51 | 3079 | A>G | Exon1 |
|  | SNP52 | 3075 | C>G | Exon1 |
|  | SNP53 | 3057 | A>G | Exon1 |
|  | SNP54 | 3051 | T>C | Exon1 |
|  | SNP55 | 3022 | T>A | Exon1 |
|  | SNP56 | 2995 | G>A | Exon1 |
|  | SNP57 | 2988 | T>G | Exon1 |
|  | SNP58 | 2558 | A>G | Intron in 5'UTR |
|  | SNP59 | 2448 | A>G | Intron in 5'UTR |
|  | SNP60 | 2375 | T>C | Intron in 5'UTR |
|  | SNP61 | 2366 | T>G | Intron in 5'UTR |
|  | SNP62 | 2205 | A>G | 5'UTR |
|  | SNP63 | 2151 | T>C | 5'UTR |
|  | SNP64 | 1955 | T>C | Promoter |
|  | SNP65 | 1943 | T>C | Promoter |
|  | SNP66 | 1917 | A>T | Promoter |
|  | SNP67 | 1914 | T>A | Promoter |
|  | SNP68 | 1897 | C>T | Promoter |
|  | SNP69 | 1746 | C>T | Promoter |
|  | SNP70 | 1721 | C>A | Promoter |
|  | SNP71 | 1717 | A>G | Promoter |
|  | SNP72 | 1626 | C>T | Promoter |
|  | SNP73 | 1593 | C>G | Promoter |
|  | SNP74 | 1568 | A>G | Promoter |
|  | SNP75 | 1560 | A>G | Promoter |
|  | SNP76 | 1547 | A>G | Promoter |
|  | SNP77 | 1409 | T>C | Promoter |
|  | SNP78 | 1329 | G>C | Promoter |
|  | SNP79 | 1298 | T>C | Promoter |
|  | SNP80 | 1294 | A>G | Promoter |
|  | SNP81 | 1115 | C>T | Promoter |
|  | SNP82 | 1011 | G>A | Promoter |
|  | SNP83 | 866 | T>G | Promoter |
|  | SNP84 | 863 | A>G | Promoter |
|  | SNP85 | 860 | C>A | Promoter |
|  | SNP86 | 833 | G>A | Promoter |
|  | SNP87 | 822 | T>A | Promoter |
|  | SNP88 | 811 | T>A | Promoter |
|  | SNP89 | 807 | A>G | Promoter |
|  | SNP90 | 793 | A>G | Promoter |
|  | SNP91 | 791 | T>C | Promoter |
|  | SNP92 | 787 | C>T | Promoter |
|  | SNP93 | 774 | C>G | Promoter |
|  | SNP94 | 763 | G>A | Promoter |
|  | SNP95 | 741 | G>C | Promoter |
|  | SNP96 | 710 | A>G | Promoter |
|  | SNP97 | 700 | T>C | Promoter |
|  | SNP98 | 695 | A>T | Promoter |
|  | SNP99 | 654 | C>A | Promoter |
|  | SNP100 | 649 | G>C | Promoter |
|  | SNP101 | 560 | C>A | Promoter |
|  | SNP102 | 556 | T>C | Promoter |
|  | SNP103 | 477 | A>G | Promoter |
|  | SNP104 | 442 | C>G | Promoter |
|  | SNP105 | 419 | G>T | Promoter |
|  | SNP106 | 336 | T>A | Promoter |
|  | SNP107 | 325 | T>G | Promoter |
|  | SNP108 | 276 | T>C | Promoter |
|  | SNP109 | 241 | G>A | Promoter |
|  | SNP110 | 197 | T>G | Promoter |
|  | SNP111 | 75 | A>G | Promoter |
| *Pto-SPL20* |  |  |  |  |
|  | SNP1 | 4333 | T>C | Flanking |
|  | SNP2 | 4312 | C>T | Flanking |
|  | SNP3 | 4275 | C>A | Flanking |
|  | SNP4 | 4261 | G>C | Flanking |
|  | SNP5 | 4252 | C>T | Flanking |
|  | SNP6 | 4235 | C>T | Flanking |
|  | SNP7 | 4122 | A>G | Flanking |
|  | SNP8 | 4065 | C>A | Flanking |
|  | SNP9 | 4028 | C>A | Flanking |
|  | SNP10 | 4012 | A>T | Flanking |
|  | SNP11 | 3992 | A>C | Flanking |
|  | SNP12 | 3964 | G>A | Flanking |
|  | SNP13 | 3924 | A>C | Flanking |
|  | SNP14 | 3942 | T>C | Flanking |
|  | SNP15 | 3835 | G>A | 3'UTR |
|  | SNP16 | 3833 | A>G | 3'UTR |
|  | SNP17 | 3789 | C>A | 3'UTR |
|  | SNP18 | 3755 | C>T | 3'UTR |
|  | SNP19 | 3740 | A>G | 3'UTR |
|  | SNP20 | 3739 | T>C | 3'UTR |
|  | SNP21 | 3452 | C>G | Exon2 |
|  | SNP22 | 3443 | A>G | Exon2 |
|  | SNP23 | 3294 | A>G | Intron |
|  | SNP24 | 3263 | T>G | Intron |
|  | SNP25 | 3256 | G>A | Intron |
|  | SNP26 | 3211 | T>C | Intron |
|  | SNP27 | 3195 | T>C | Intron |
|  | SNP28 | 3189 | T>C | Intron |
|  | SNP29 | 3142 | C>T | Intron |
|  | SNP30 | 3103 | A>T | Intron |
|  | SNP31 | 2914 | C>T | Intron |
|  | SNP32 | 2853 | A>C | Intron |
|  | SNP33 | 2751 | C>T | Intron |
|  | SNP34 | 2709 | T>A | Intron |
|  | SNP35 | 2562 | C>T | Intron |
|  | SNP36 | 2554 | T>C | Intron |
|  | SNP37 | 2523 | T>C | Intron |
|  | SNP38 | 2514 | G>A | Intron |
|  | SNP39 | 2482 | G>T | Intron |
|  | SNP40 | 2444 | G>T | Intron |
|  | SNP41 | 2432 | A>T | Intron |
|  | SNP42 | 2398 | T>C | Intron |
|  | SNP43 | 2382 | G>A | Intron |
|  | SNP44 | 2371 | A>G | Intron |
|  | SNP45 | 2367 | A>C | Intron |
|  | SNP46 | 2320 | T>G | Exon1 |
|  | SNP47 | 2184 | A>G | Exon1 |
|  | SNP48 | 2169 | C>T | Exon1 |
|  | SNP49 | 2132 | A>G | Exon1 |
|  | SNP50 | 1999 | T>A | 5'UTR |
|  | SNP51 | 1833 | A>T | 5'UTR |
|  | SNP52 | 1756 | C>T | Promoter |
|  | SNP53 | 1754 | G>A | Promoter |
|  | SNP54 | 1744 | G>A | Promoter |
|  | SNP55 | 1728 | A>G | Promoter |
|  | SNP56 | 1726 | A>G | Promoter |
|  | SNP57 | 1721 | T>C | Promoter |
|  | SNP58 | 1681 | C>T | Promoter |
|  | SNP59 | 1675 | C>G | Promoter |
|  | SNP60 | 1554 | A>T | Promoter |
|  | SNP61 | 1538 | A>T | Promoter |
|  | SNP62 | 1530 | G>A | Promoter |
|  | SNP63 | 1529 | A>C | Promoter |
|  | SNP64 | 1466 | A>G | Promoter |
|  | SNP65 | 1377 | T>A | Promoter |
|  | SNP66 | 1362 | T>A | Promoter |
|  | SNP67 | 1352 | A>T | Promoter |
|  | SNP68 | 1338 | A>G | Promoter |
|  | SNP69 | 1319 | A>G | Promoter |
|  | SNP70 | 1298 | G>T | Promoter |
|  | SNP71 | 1273 | T>C | Promoter |
|  | SNP72 | 1088 | T>C | Promoter |
|  | SNP73 | 1042 | G>A | Promoter |
|  | SNP74 | 952 | A>C | Promoter |
|  | SNP75 | 511 | T>C | Promoter |
|  | SNP76 | 507 | T>C | Promoter |
|  | SNP77 | 441 | A>G | Promoter |
|  | SNP78 | 436 | T>C | Promoter |
|  | SNP79 | 435 | G>A | Promoter |
|  | SNP80 | 427 | A>G | Promoter |
|  | SNP81 | 394 | G>A | Promoter |
|  | SNP82 | 374 | G>A | Promoter |
|  | SNP83 | 362 | T>C | Promoter |
|  | SNP84 | 319 | C>A | Promoter |
|  | SNP85 | 280 | T>A | Promoter |
|  | SNP86 | 224 | G>T | Promoter |
|  | SNP87 | 214 | C>A | Promoter |
|  | SNP88 | 32 | C>T | Promoter |
|  | SNP89 | 27 | C>T | Promoter |
| *Pto-SPL25* |  |  |  |  |
|  | SNP1 | 4667 | T>C | Flanking |
|  | SNP2 | 4198 | G>C | Flanking |
|  | SNP3 | 4194 | G>T | Flanking |
|  | SNP4 | 4177 | T>A | Flanking |
|  | SNP5 | 4157 | G>A | 3' UTR |
|  | SNP6 | 4086 | G>A | 3' UTR |
|  | SNP7 | 4074 | A>G | 3' UTR |
|  | SNP8 | 3890 | A>C | Exon2 |
|  | SNP9 | 3863 | A>G | Exon2 |
|  | SNP10 | 3842 | A>G | Exon2 |
|  | SNP11 | 3775 | G>C | Exon2 |
|  | SNP12 | 3646 | G>A | Intron |
|  | SNP13 | 3612 | A>G | Intron |
|  | SNP14 | 3610 | A>C | Intron |
|  | SNP15 | 3502 | A>G | Intron |
|  | SNP16 | 3440 | T>A | Intron |
|  | SNP17 | 3369 | T>A | Intron |
|  | SNP18 | 3348 | A>G | Intron |
|  | SNP19 | 3341 | C>T | Intron |
|  | SNP20 | 3332 | T>C | Intron |
|  | SNP21 | 3303 | A>G | Intron |
|  | SNP22 | 3293 | C>G | Intron |
|  | SNP23 | 3280 | A>C | Intron |
|  | SNP24 | 3240 | T>C | Intron |
|  | SNP25 | 3211 | C>T | Intron |
|  | SNP26 | 3177 | A>G | Intron |
|  | SNP27 | 3146 | C>T | Intron |
|  | SNP28 | 3087 | T>C | Intron |
|  | SNP29 | 3025 | C>T | Intron |
|  | SNP30 | 2929 | C>T | Intron |
|  | SNP31 | 2904 | G>A | Intron |
|  | SNP32 | 2859 | A>C | Intron |
|  | SNP33 | 2483 | G>T | Exon1 |
|  | SNP34 | 2417 | C>T | Exon1 |
|  | SNP35 | 2355 | A>G | Exon1 |
|  | SNP36 | 2353 | T>C | Exon1 |
|  | SNP37 | 2236 | C>T | Exon1 |
|  | SNP38 | 2220 | C>G | Exon1 |
|  | SNP39 | 2131 | C>A | Exon1 |
|  | SNP40 | 2136 | G>A | 5'UTR |
|  | SNP41 | 2102 | A>G | 5'UTR |
|  | SNP42 | 2078 | C>T | 5'UTR |
|  | SNP43 | 2044 | G>A | 5'UTR |
|  | SNP44 | 1957 | G>A | Promoter |
|  | SNP45 | 1869 | T>A | Promoter |
|  | SNP46 | 1752 | A>T | Promoter |
|  | SNP47 | 1678 | A>G | Promoter |
|  | SNP48 | 1599 | G>A | Promoter |
|  | SNP49 | 1209 | T>A | Promoter |
|  | SNP50 | 1172 | A>T | Promoter |
|  | SNP51 | 1142 | C>A | Promoter |
|  | SNP52 | 1131 | C>T | Promoter |
|  | SNP53 | 1083 | C>T | Promoter |
|  | SNP54 | 872 | C>T | Promoter |
|  | SNP55 | 848 | A>C | Promoter |
|  | SNP56 | 740 | T>C | Promoter |
|  | SNP57 | 370 | G>A | Promoter |
|  | SNP58 | 298 | C>G | Promoter |
|  | SNP59 | 251 | C>G | Promoter |
|  | SNP60 | 244 | C>A | Promoter |
|  | SNP61 | 236 | A>G | Promoter |
|  | SNP62 | 205 | A>T | Promoter |
|  | SNP63 | 185 | C>T | Promoter |
|  | SNP64 | 90 | G>A | Promoter |
|  | SNP65 | 23 | C>T | Promoter |
|  | SNP66 | 19 | C>T | Promoter |

^a^SNP position: The SNP position in genes (bp).

^b^flanking: 500bp downstream of 3′ UTR of *Pto-SPL15*, *Pto-SPL20*, and *Pto-SPL25*.
