# Supplementary material for: Association Studies in Populus tomentosa Reveal the Genetic Interactions of Pto-MIR156c and Its Targets in Wood Formation
Source: Front Plant Sci. 2016 Aug 3;7:1159. doi: 10.3389/fpls.2016.01159 (PMC4971429; doi:10.3389/fpls.2016.01159)
Supplement: Table S4 — Details of significant SNPs within Pto-MIR156c and the three potential targets of Pto-miR156c associated with growth and wood properties in an association population of P. tomentosa. [file Table4.DOC]

**Table S4** Details of significant SNPs within *Pto-MIR156c* and the three potential targets of *Pto-miR156c* associated with growth and wood properties in an association population of *P. tomentosa*.

| **Gene** | **Traits** | **SNP locus** | **Position** | **Additive effect** | **Dominant effect** | ***R^2^* (%)** | ***P*-value** | **Q-value** |
| --- | --- | --- | --- | --- | --- | --- | --- | --- |
| *Pto-MIR156c* |  |  |  |  |  |  |  |  |
|  | HC | SNP4 | Flanking region | - | -4.15 | 5.05 | 1.41E-03 | 6.32E-02 |
|  | HEC | SNP9 | Pre-mature region | - | -3.73 | 4.58 | 2.25E-03 | 8.12E-02 |
|  | HEC | SNP10 | Pre-mature region | - | -7.50 | 2.79 | 5.61E-03 | 1.38E-02 |
| *Pto-SPL15* |  |  |  |  |  |  |  |  |
|  | DBH | SNP8 | 3′ UTR | 2.14 | 0.52 | 13.18 | 3.12E-05 | 1.71E-02 |
|  | V | SNP8 | 3′ UTR | 29.59 | 1.91 | 6.80 | 7.70E-04 | 4.28E-02 |
|  | DBH | SNP28 | Intron | 4.85 | 2.63 | 7.89 | 4.85E-04 | 3.12E-02 |
|  | FW | SNP37 | Intron | 0.06 | 1.57 | 4.93 | 2.01E-03 | 7.80E-02 |
|  | FW | SNP63 | 5′ UTR | 1.20 | 1.97 | 4.23 | 2.87E-03 | 9.22E-02 |
|  | DBH | SNP64 | Promoter | 2.83 | 5.48 | 12.69 | 4.05E-05 | 1.71E-02 |
|  | V | SNP64 | Promoter | 24.53 | 45.81 | 5.74 | 1.33E-03 | 6.06E-02 |
|  | DBH | SNP66 | Promoter | 2.03 | -3.46 | 13.40 | 1.62E-04 | 1.95E-02 |
|  | V | SNP66 | Promoter | - | -24.76 | 7.92 | 2.13E-03 | 8.04E-02 |
|  | DBH | SNP68 | Promoter | - | -5.87 | 9.61 | 1.04E-03 | 5.37E-02 |
|  | DBH | SNP69 | Promoter | 7.11 | 4.74 | 8.22 | 4.09E-04 | 3.11E-02 |
|  | FW | SNP70 | Promoter | 0.93 | 1.59 | 5.98 | 1.27E-03 | 5.94E-02 |
|  | DBH | SNP81 | Promoter | 2.00 | 15.92 | 10.73 | 9.58E-04 | 5.05E-02 |
|  | V | SNP81 | Promoter | - | 40.90 | 15.52 | 8.91E-05 | 1.95E-02 |
|  | DBH | SNP82 | Promoter | 2.71 | - | 13.42 | 1.61E-04 | 1.95E-02 |
|  | V | SNP82 | Promoter | 24.62 | - | 7.12 | 3.11E-03 | 9.54E-02 |
|  | DBH | SNP89 | Promoter | 3.28 | - | 19.32 | 8.28E-06 | 1.70E-02 |
|  | V | SNP89 | Promoter | 31.44 | - | 11.23 | 4.56E-04 | 3.12E-02 |
|  | CC | SNP106 | Promoter | 3.60 | -1.30 | 11.44 | 6.65E-05 | 1.71E-02 |
|  | HEC | SNP106 | Promoter | 3.59 | 2.69 | 7.59 | 4.81E-04 | 3.12E-02 |
|  | FW | SNP111 | Promoter | 0.10 | 1.37 | 9.02 | 2.50E-04 | 2.34E-02 |
| *Pto-SPL20* |  |  |  |  |  |  |  |  |
|  | DBH | SNP2 | Flanking region | 6.39 | 4.74 | 8.42 | 3.71E-04 | 2.93E-02 |
|  | DBH | SNP7 | Flanking region | 2.58 | - | 8.69 | 1.75E-03 | 7.38E-02 |
|  | HEC | SNP8 | Flanking region | 5.90 | 0.60 | 10.36 | 1.27E-04 | 1.95E-02 |
|  | HC | SNP9 | Flanking region | 3.06 | -4.32 | 7.06 | 2.75E-03 | 8.97E-02 |
|  | V | SNP21 | Exon | 28.92 | -38.07 | 11.13 | 4.45E-04 | 3.12E-02 |
|  | V | SNP23 | Intron | 32.05 | -37.90 | 11.60 | 3.53E-04 | 2.90E-02 |
|  | DBH | SNP30 | Intron | 8.87 | -7.26 | 7.97 | 4.66E-04 | 3.12E-02 |
|  | V | SNP30 | Intron | 4.01 | -2.97 | 4.31 | 2.72E-03 | 8.97E-02 |
|  | FW | SNP41 | Intron | 0.62 | -7.26 | 4.24 | 3.04E-03 | 9.47E-02 |
|  | DBH | SNP46 | Exon | - | -5.65 | 9.49 | 1.10E-03 | 5.41E-02 |
|  | V | SNP46 | Exon | - | -26.57 | 7.19 | 3.02E-03 | 9.47E-02 |
|  | FW | SNP73 | Exon | 0.80 | 1.22 | 5.25 | 1.72E-03 | 7.38E-02 |
|  | FW | SNP88 | Exon | 0.81 | 2.19 | 6.84 | 7.66E-04 | 4.28E-02 |
|  | FW | SNP89 | Exon | 0.87 | 1.92 | 4.54 | 2.45E-03 | 8.55E-02 |
| *Pto-SPL25* |  |  |  |  |  |  |  |  |
|  | DBH | SNP12 | Intron | 2.44 | - | 9.67 | 1.10E-03 | 5.41E-02 |
|  | DBH | SNP14 | Intron | 2.99 | - | 15.59 | 5.32E-05 | 1.71E-02 |
|  | V | SNP14 | Intron | 27.62 | - | 8.21 | 1.86E-03 | 7.64E-02 |
|  | DBH | SNP15 | Intron | 2.27 | - | 8.27 | 2.15E-03 | 8.04E-02 |
|  | DBH | SNP16 | Intron | 2.53 | - | 9.16 | 2.23E-03 | 8.12E-02 |
|  | DBH | SNP22 | Intron | 1.83 | -3.20 | 11.59 | 5.57E-04 | 3.47E-02 |
|  | DBH | SNP27 | Intron | 2.45 | -2.06 | 7.86 | 2.60E-03 | 8.92E-02 |
|  | DBH | SNP28 | Intron | 2.80 | -2.98 | 12.84 | 3.05E-04 | 2.61E-02 |
|  | V | SNP28 | Intron | 28.34 | - | 8.32 | 2.32E-03 | 8.21E-02 |
|  | DBH | SNP29 | Intron | 2.69 | - | 12.37 | 2.95E-04 | 2.61E-02 |
|  | DBH | SNP30 | Intron | 2.78 | - | 13.50 | 1.61E-04 | 1.95E-02 |
|  | FW | SNP30 | Intron | - | 3.85 | 13.33 | 1.64E-04 | 1.95E-02 |
|  | DBH | SNP31 | Intron | 2.81 | - | 13.20 | 1.80E-04 | 1.95E-02 |
|  | V | SNP31 | Intron | 27.42 | - | 8.04 | 2.01E-03 | 7.80E-02 |
|  | DBH | SNP39 | Exon | 2.81 | - | 13.20 | 1.80E-04 | 1.95E-02 |
|  | V | SNP39 | Exon | 27.42 | - | 8.04 | 2.01E-03 | 7.80E-02 |
|  | DBH | SNP41 | 5′ UTR | 3.23 | - | 15.70 | 5.02E-05 | 1.71E-02 |
|  | V | SNP41 | 5′ UTR | 32.60 | - | 10.53 | 6.01E-04 | 3.63E-02 |
|  | DBH | SNP42 | 5′ UTR | 3.22 | - | 15.34 | 6.05E-05 | 1.71E-02 |
|  | V | SNP42 | 5′ UTR | 32.23 | - | 10.25 | 6.89E-04 | 4.05E-02 |
|  | DBH | SNP44 | Promoter | 2.70 | - | 12.89 | 2.19E-04 | 2.14E-02 |
|  | DBH | SNP45 | Promoter | 6.37 | - | 12.63 | 4.37E-05 | 1.71E-02 |
|  | V | SNP45 | Promoter | 26.34 | - | 5.90 | 1.25E-03 | 5.94E-02 |
|  | DBH | SNP46 | Promoter | 2.56 | - | 14.08 | 1.34E-04 | 1.95E-02 |
|  | V | SNP46 | Promoter | 23.02 | - | 7.23 | 3.23E-03 | 9.76E-02 |
|  | DBH | SNP47 | Promoter | 2.77 |  | 13.39 | 2.12E-04 | 2.14E-02 |
|  | DBH | SNP48 | Promoter | 0.77 | -0.14 | 10.41 | 1.52E-04 | 1.95E-02 |
|  | V | SNP48 | Promoter | 25.23 |  | 4.46 | 2.72E-03 | 8.97E-02 |
|  | DBH | SNP51 | Promoter | 2.85 |  | 14.21 | 1.13E-04 | 1.95E-02 |
|  | FW | SNP52 | Promoter | 0.30 | -0.31 | 5.45 | 1.76E-03 | 7.38E-02 |
|  | FW | SNP55 | Promoter | 1.80 | 1.58 | 16.28 | 8.12E-04 | 4.39E-02 |
