# Supplementary material for: Association Studies in Populus tomentosa Reveal the Genetic Interactions of Pto-MIR156c and Its Targets in Wood Formation
Source: Front Plant Sci. 2016 Aug 3;7:1159. doi: 10.3389/fpls.2016.01159 (PMC4971429; doi:10.3389/fpls.2016.01159)
Supplement: Table S5 — Significant haplotypes from Pto-MIR156c and its potential targets associated with growth and wood properties in the association population. [file Table5.DOC]

**Table S5** Significant haplotypes from *Pto-MIR156c* and its potential targets associated with growth and wood properties in the association population

| **Gene** | **LD Blocks** | **Significant Haplotypes** | **Haplotype**  **frequency** | **Associated traits** | ***P*-value** | ***R*^2^ (%)** | | |
| --- | --- | --- | --- | --- | --- | --- | --- | --- |
| *Pto-MIR156c* |  |  |  |  |  | |  |  |
|  | SNP1-3 | AGC | 0.12 | FW | 6.35E-03 | | 3.02 |  |
|  |  | TTA | 0.86 | DBH | 8.79E-03 | | 1.16 |  |
|  |  |  |  | FW | 5.87E-03 | | 3.02 |  |
|  | SNP5-8 | AC | 0.13 | FW | 4.12E-03 | | 2.79 |  |
|  |  |  |  | HEC | 2.06E-03 | | 2.89 |  |
|  |  | GT | 0.86 | FW | 4.65E-03 | | 2.79 |  |
|  |  |  |  | HEC | 6.07E-03 | | 2.89 |  |
|  | SNP9-10 | AA | 0.51 | CC | 4.17E-03 | | 1.12 |  |
|  |  |  |  | HEC | 3.53E-03 | | 0.15 |  |
|  |  | GG | 0.49 | HEC | 1.81E-03 | | 0.15 |  |
|  | SNP11-17 | TGTTA | 0.86 | DBH | 8.96E-03 | | 4.35 |  |
|  |  |  |  | FW | 5.54E-03 | | 2.04 |  |
| *Pto-SPL15* |  |  |  |  |  | |  |  |
|  | SNP5-7 | ATA | 0.13 | DBH | 4.65E-03 | | 5.41 |  |
|  |  |  |  | FW | 3.92E-03 | | 3.10 |  |
|  |  |  |  | V | 8.44E-03 | | 6.19 |  |
|  |  | GAG | 0.86 | DBH | 8.79E-03 | | 5.41 |  |
|  |  |  |  | FW | 5.87E-03 | | 3.10 |  |
|  | SNP10-22 | AAAACCA | 0.86 | DBH | 9.14E-03 | | 0.57 |  |
|  |  |  |  | FW | 5.91E-03 | | 0.68 |  |
|  | SNP29-43 | ACCATCAAT | 0.86 | DBH | 9.57E-03 | | 9.12 |  |
|  |  |  |  | FW | 6.09E-03 | | 2.54 |  |
|  |  | GTTGCTCGA | 0.10 | FW | 6.08E-03 | | 2.54 |  |
|  | SNP46-48 | AAC | 0.05 | FL | 9.90E-03 | | 9.10 |  |
|  |  |  |  | FW | 9.01E-03 | | 8.90 |  |
|  |  | GGG | 0.94 | FW | 9.84E-03 | | 8.90 |  |
|  | SNP83-85 | GGA | 0.11 | DBH | 1.59E-03 | | 1.82 |  |
|  |  |  |  | V | 3.38E-03 | | 2.04 |  |
|  | SNP86-88 | AAA | 0.14 | HEC | 4.06E-03 | | 7.32 |  |
|  | SNP90-93 | ATCC | 0.84 | HEC | 9.59E-03 | | 2.76 |  |
|  |  | GCTG | 0.14 | HEC | 1.61E-03 | | 2.76 |  |
|  | SNP97-98 | CTATC | 0.06 | HEC | 8.49E-03 | | 0.66 |  |
|  |  | TA | 0.94 | HEC | 6.05E-03 | | 0.66 |  |
|  | SNP101-102 | AC | 0.08 | MFA | 3.16E-02 | | 13.59 |  |
|  |  | CT | 0.92 | MFA | 4.37E-03 | | 13.59 |  |
| *Pto-SPL20* |  |  |  |  |  | |  |  |
|  | SNP3-6 | ACTT | 0.06 | DBH | 5.82E-03 | | 4.79 |  |
|  |  | CGCC | 0.87 | DBH | 4.90E-04 | | 4.79 |  |
|  |  |  |  | HEC | 2.18E-03 | | 1.28 |  |
|  |  |  |  | MFA | 3.05E-03 | | 1.71 |  |
|  |  |  |  | V | 1.53E-03 | | 2.64 |  |
|  |  | CGTC | 0.06 | MFA | 8.95E-03 | | 1.71 |  |
|  | SNP18-19 | TG | 0.11 | CC | 8.22E-03 | | 1.21 |  |
|  | SNP26-29 | CCCT | 0.12 | FW | 6.12E-03 | | 3.87 |  |
|  |  | TTTC | 0.87 | FW | 9.24E-03 | | 3.87 |  |
|  | SNP32-36 | ACTCT | 0.86 | DBH | 8.96E-03 | | 4.73 |  |
|  |  |  |  | FW | 5.56E-03 | | 1.54 |  |
|  |  | CTATC | 0.12 | FW | 9.57E-03 | | 1.54 |  |
|  | SNP40-41 | GA | 0.85 | FW | 5.53E-03 | | 2.77 |  |
|  |  | TT | 0.13 | FW | 5.77E-03 | | 2.77 |  |
|  |  |  |  | HEC | 2.59E-03 | | 6.32 |  |
|  | SNP52-57 | TAAGGC | 0.06 | CC | 9.30E-03 | | 7.67 |  |
|  | SNP65-69 | TTAAA | 0.88 | DBH | 5.51E-03 | | 2.73 |  |
|  | SNP70-74 | GTTGA | 0.86 | DBH | 8.95E-03 | | 3.50 |  |
|  |  |  |  | FW | 5.55E-03 | | 1.65 |  |
|  | SNP86-89 | GCCC | 0.86 | DBH | 8.87E-03 | | 6.18 |  |
|  |  |  |  | FW | 5.85E-03 | | 4.55 |  |
|  |  | TATT | 0.10 | FW | 2.40E-03 | | 4.55 |  |
|  |  |  |  | HEC | 1.17E-03 | | 1.71 |  |
| *Pto-SPL25* |  |  |  |  |  | |  |  |
|  | SNP5-7 | AAG | 0.10 | DBH | 4.13E-03 | | 6.46 |  |
|  |  |  |  | FW | 7.85E-03 | | 3.09 |  |
|  |  |  |  | HEC | 2.12E-03 | | 7.17 |  |
|  |  |  |  | V | 7.86E-03 | | 5.55 |  |
|  |  | GGA | 0.86 | HEC | 9.65E-03 | | 7.17 |  |
|  | SNP17-18 | AG | 0.08 | DBH | 2.25E-03 | | 4.50 |  |
|  |  |  |  | V | 5.75E-03 | | 4.76 |  |
|  | SNP34-73 | CCGG | 0.83 | FW | 7.26E-03 | | 3.14 |  |
|  |  |  |  | V | 7.58E-03 | | 8.35 |  |
|  |  | TGAA | 0.09 | DBH | 1.23E-03 | | 12.45 |  |
|  |  |  |  | V | 4.57E-03 | | 8.35 |  |
|  | SNP57-63 | AGAGTT | 0.12 | CC | 5.69E-03 | | 2.20 |  |
|  |  | GCCAAC | 0.80 | DBH | 5.79E-03 | | 7.00 |  |
|  |  |  |  | FW | 2.54E-03 | | 21.73 |  |
|  |  |  |  | V | 6.37E-03 | | 29.47 |  |
