# Supplementary material for: Association Studies in Populus tomentosa Reveal the Genetic Interactions of Pto-MIR156c and Its Targets in Wood Formation
Source: Front Plant Sci. 2016 Aug 3;7:1159. doi: 10.3389/fpls.2016.01159 (PMC4971429; doi:10.3389/fpls.2016.01159)
Supplement: Table S6 — The SNP pairs and their main effects detected from Pto-MIR156c and the three potential targets of Pto-miR156c under an epistasis model in the association population of P. tomentosa. [file Table6.DOC]

**Table S6** The SNP pairs and their main effects detected from *Pto-MIR156c* and the three potential targets of *Pto-miR156c* under an epistasis model in the association population of *P. tomentosa*.

| **Traits** | **Attribute A** | **Single effect (%)** | **Attribute B** | **Single effect (% )** | **Effect of interaction (%)** | **Information gain (%)** |
| --- | --- | --- | --- | --- | --- | --- |
| DBH |  |  |  |  |  |  |
|  | Pto-MIR156c-SNP9 | 1.18 | Pto-SPL20-SNP13 | 1.19 | 1.14 | -1.23 |
|  | Pto-MIR156c-SNP9 | 1.18 | Pto-SPL20-SNP39 | 1.63 | 1.63 | -1.18 |
|  | Pto-MIR156c-SNP9 | 1.18 | Pto-SPL25-SNP66 | 3.53 | 3.48 | -1.23 |
|  | Pto-SPL20-SNP13 | 1.19 | Pto-SPL25-SNP66 | 3.53 | 3.48 | -1.24 |
|  | Pto-SPL20-SNP39 | 1.63 | Pto-SPL25-SNP66 | 3.00 | 8.41 | 3.26 |
| H |  |  |  |  |  |  |
|  | Pto-SPL20-SNP1 | 0.13 | Pto-SPL15-SNP26 | 3.73 | 0.28 | -3.58 |
|  | Pto-SPL20-SNP1 | 0.13 | Pto-SPL15-SNP71 | 1.23 | 1.81 | 0.45 |
|  | Pto-SPL20-SNP1 | 0.13 | Pto-SPL15-SNP101 | 2.01 | 2.60 | 0.46 |
|  | Pto-SPL20-SNP6 | 1.14 | Pto-SPL15-SNP26 | 3.73 | 1.05 | -3.82 |
|  | Pto-SPL20-SNP6 | 1.14 | Pto-SPL15-SNP71 | 1.23 | 4.03 | 1.67 |
|  | Pto-SPL20-SNP6 | 1.14 | Pto-SPL15-SNP101 | 2.01 | 2.30 | -0.85 |
|  | Pto-SPL20-SNP19 | 0.08 | Pto-SPL15-SNP26 | 3.73 | 0.52 | -3.29 |
|  | Pto-SPL20-SNP19 | 0.08 | Pto-SPL15-SNP71 | 1.23 | 1.63 | 0.32 |
|  | Pto-SPL20-SNP19 | 0.08 | Pto-SPL15-SNP101 | 2.01 | 0.78 | -1.31 |
| V |  |  |  |  |  |  |
|  | Pto-SPL20-SNP14 | 1.60 | Pto-SPL15-SNP92 | 2.33 | 1.45 | -2.47 |
|  | Pto-SPL20-SNP14 | 1.60 | Pto-SPL15-SNP97 | 0.11 | 2.30 | 0.59 |
|  | Pto-SPL20-SNP14 | 1.60 | Pto-SPL15-SNP104 | 2.57 | 2.12 | -2.05 |
|  | Pto-SPL20-SNP49 | 1.27 | Pto-SPL15-SNP92 | 2.33 | 3.48 | -0.12 |
|  | Pto-SPL20-SNP49 | 1.27 | Pto-SPL15-SNP97 | 0.11 | 0.11 | -1.27 |
|  | Pto-SPL20-SNP49 | 1.27 | Pto-SPL15-SNP104 | 2.57 | 2.30 | -1.54 |
|  | Pto-SPL20-SNP64 | 0.42 | Pto-SPL15-SNP92 | 2.33 | 0.90 | -1.85 |
|  | Pto-SPL20-SNP64 | 0.42 | Pto-SPL15-SNP97 | 0.11 | 1.58 | 1.05 |
|  | Pto-SPL20-SNP64 | 0.42 | Pto-SPL15-SNP104 | 2.57 | 1.45 | -1.54 |
| MFA |  |  |  |  |  |  |
|  | Pto-SPL20-SNP14 | 1.73 | Pto-SPL25-SNP2 | 1.18 | 0.62 | -2.29 |
|  | Pto-SPL20-SNP14 | 1.73 | Pto-SPL25-SNP62 | 1.18 | 0.24 | -2.68 |
|  | Pto-SPL20-SNP14 | 1.73 | Pto-SPL15-SNP54 | 0.24 | 0.55 | -1.42 |
|  | Pto-SPL20-SNP14 | 1.73 | Pto-SPL15-SNP58 | 0.95 | 0.13 | -2.55 |
|  | Pto-SPL20-SNP14 | 1.73 | Pto-SPL15-SNP104 | 5.74 | 0 | -7.47 |
|  | Pto-SPL25-SNP2 | 1.18 | Pto-SPL15-SNP54 | 0.24 | 0.52 | -0.90 |
|  | Pto-SPL25-SNP2 | 1.18 | Pto-SPL15-SNP58 | 0.95 | 0.05 | -2.08 |
|  | Pto-SPL25-SNP2 | 1.18 | Pto-SPL15-SNP104 | 5.74 | 2.38 | -4.55 |
|  | Pto-SPL25-SNP62 | 1.18 | Pto-SPL15-SNP54 | 0.24 | 0.87 | -0.56 |
|  | Pto-SPL25-SNP62 | 1.18 | Pto-SPL15-SNP58 | 0.95 | 0.63 | -1.51 |
|  | Pto-SPL25-SNP62 | 1.18 | Pto-SPL15-SNP104 | 5.74 | 0.56 | -6.36 |
| FL |  |  |  |  |  |  |
|  | Pto-MIR156c-SNP4 | 2.03 | Pto-SPL20-SNP1 | 1.01 | 3.50 | 0.46 |
|  | Pto-MIR156c-SNP4 | 2.03 | Pto-SPL15-SNP104 | 5.74 | 3.48 | -4.29 |
|  | Pto-SPL20-SNP1 | 1.01 | Pto-SPL15-SNP104 | 5.74 | 3.48 | -3.27 |
| FW |  |  |  |  |  |  |
|  | Pto-MIR156c-SNP9 | 1.18 | Pto-SPL20-SNP47 | 1.22 | 2.38 | -0.02 |
|  | Pto-MIR156c-SNP9 | 1.18 | Pto-SPL25-SNP55 | 7.45 | 8.70 | 0.07 |
|  | Pto-MIR156c-SNP9 | 1.18 | Pto-SPL15-SNP75 | 2.35 | 0.33 | -3.20 |
|  | Pto-MIR156c-SNP9 | 1.18 | Pto-SPL15-SNP102 | 0.47 | 0.33 | -1.32 |
|  | Pto-MIR156c-SNP10 | 0 | Pto-SPL20-SNP47 | 1.22 | 2.38 | 1.16 |
|  | Pto-MIR156c-SNP10 | 0 | Pto-SPL25-SNP55 | 7.45 | 4.70 | -2.75 |
|  | Pto-MIR156c-SNP10 | 0 | Pto-SPL15-SNP75 | 2.35 | 0.75 | -1.60 |
|  | Pto-MIR156c-SNP10 | 0 | Pto-SPL15-SNP102 | 0.47 | 0.09 | -0.38 |
|  | Pto-SPL20-SNP47 | 1.22 | Pto-SPL25-SNP55 | 7.45 | 2.76 | -5.91 |
|  | Pto-SPL20-SNP47 | 1.22 | Pto-SPL15-SNP75 | 2.35 | 0.08 | -3.49 |
|  | Pto-SPL20-SNP47 | 1.22 | Pto-SPL15-SNP102 | 0.47 | 0.24 | -1.45 |
|  | Pto-SPL25-SNP55 | 7.45 | Pto-SPL15-SNP75 | 2.35 | 1.15 | -8.66 |
|  | Pto-SPL25-SNP55 | 7.45 | Pto-SPL15-SNP102 | 0.47 | 0.78 | -7.15 |
| CC |  |  |  |  |  |  |
|  | Pto-MIR156c-SNP15 | 2.01 | Pto-SPL20-SNP5 | 2.40 | 0.03 | -4.39 |
|  | Pto-MIR156c-SNP15 | 2.01 | Pto-SPL15-SNP16 | 0.08 | 3.46 | 1.37 |
|  | Pto-MIR156c-SNP15 | 2.01 | Pto-SPL15-SNP58 | 0.95 | 0.56 | -2.39 |
|  | Pto-MIR156c-SNP15 | 2.01 | Pto-SPL15-SNP68 | 1.18 | 2.30 | -0.89 |
|  | Pto-MIR156c-SNP15 | 2.01 | Pto-SPL15-SNP93 | 3.60 | 4.84 | -0.77 |
|  | Pto-SPL20-SNP5 | 2.40 | Pto-SPL15-SNP16 | 0.08 | 0.24 | -2.25 |
|  | Pto-SPL20-SNP5 | 2.40 | Pto-SPL15-SNP58 | 0.95 | 0.03 | -3.32 |
|  | Pto-SPL20-SNP5 | 2.40 | Pto-SPL15-SNP68 | 1.18 | 0.01 | -3.58 |
|  | Pto-SPL20-SNP5 | 2.40 | Pto-SPL15-SNP93 | 3.60 | 3.26 | -2.74 |
| HC |  |  |  |  |  |  |
|  | Pto-SPL20-SNP23 | 2.34 | Pto-SPL25-SNP9 | 2.01 | 1.26 | -3.09 |
|  | Pto-SPL20-SNP23 | 2.34 | Pto-SPL15-SNP63 | 1.19 | 1.26 | -2.27 |
|  | Pto-SPL20-SNP37 | 1.96 | Pto-SPL25-SNP9 | 2.01 | 1.11 | -2.87 |
|  | Pto-SPL20-SNP37 | 1.96 | Pto-SPL15-SNP63 | 1.19 | 1.11 | -2.05 |
|  | Pto-SPL20-SNP38 | 0.88 | Pto-SPL25-SNP9 | 2.01 | 0.31 | -2.58 |
|  | Pto-SPL20-SNP38 | 0.88 | Pto-SPL15-SNP63 | 1.19 | 0.31 | -1.76 |
|  | Pto-SPL20-SNP62 | 0.17 | Pto-SPL25-SNP9 | 2.01 | 3.82 | 1.64 |
|  | Pto-SPL20-SNP62 | 0.17 | Pto-SPL15-SNP63 | 1.19 | 3.82 | 2.46 |
|  | Pto-SPL25-SNP9 | 2.01 | Pto-SPL15-SNP63 | 1.19 | 2.38 | -0.82 |
| HEC |  |  |  |  |  |  |
|  | Pto-MIR156c-SNP9 | 1.18 | Pto-SPL20-SNP20 | 0.56 | 1.18 | -0.56 |
|  | Pto-MIR156c-SNP9 | 1.18 | Pto-SPL20-SNP24 | 4.70 | 5.81 | -0.07 |
|  | Pto-MIR156c-SNP9 | 1.18 | Pto-SPL25-SNP24 | 2.58 | 1.65 | -2.10 |
|  | Pto-MIR156c-SNP9 | 1.18 | Pto-SPL25-SNP51 | 2.97 | 1.26 | -2.89 |
|  | Pto-MIR156c-SNP9 | 1.18 | Pto-SPL15-SNP89 | 3.04 | 1.99 | -2.22 |
|  | Pto-SPL20-SNP20 | 0.56 | Pto-SPL25-SNP24 | 2.58 | 2.48 | -0.66 |
|  | Pto-SPL20-SNP20 | 0.56 | Pto-SPL25-SNP51 | 2.97 | 1.13 | -2.40 |
|  | Pto-SPL20-SNP20 | 0.56 | Pto-SPL15-SNP89 | 3.04 | 1.81 | -1.79 |
|  | Pto-SPL20-SNP24 | 4.70 | Pto-SPL25-SNP24 | 2.58 | 4.29 | -2.99 |
|  | Pto-SPL20-SNP24 | 4.70 | Pto-SPL25-SNP51 | 2.97 | 2.30 | -5.37 |
|  | Pto-SPL20-SNP24 | 4.70 | Pto-SPL15-SNP89 | 3.04 | 3.91 | -3.82 |
|  | Pto-SPL25-SNP24 | 2.58 | Pto-SPL15-SNP89 | 3.04 | 3.48 | -2.13 |
|  | Pto-SPL25-SNP51 | 2.97 | Pto-SPL15-SNP89 | 3.04 | 1.82 | -4.19 |
| LC |  |  |  |  |  |  |
|  | Pto-MIR156c-SNP1 | 0.37 | Pto-SPL20-SNP24 | 0 | 1.19 | 0.82 |
|  | Pto-MIR156c-SNP1 | 0.37 | Pto-SPL20-SNP43 | 3.25 | 0.66 | -2.95 |
|  | Pto-MIR156c-SNP1 | 0.37 | Pto-SPL15-SNP65 | 1.32 | 0.19 | -1.50 |
|  | Pto-MIR156c-SNP1 | 0.37 | Pto-SPL15-SNP82 | 1.46 | 0.49 | -1.33 |
|  | Pto-MIR156c-SNP1 | 0.37 | Pto-SPL15-SNP110 | 0 | 0.28 | -0.09 |
|  | Pto-SPL20-SNP24 | 0 | Pto-SPL15-SNP65 | 1.32 | 0 | -1.32 |
|  | Pto-SPL20-SNP24 | 0 | Pto-SPL15-SNP82 | 1.46 | 0.08 | -1.38 |
|  | Pto-SPL20-SNP24 | 0 | Pto-SPL15-SNP110 | 0.00 | 0.56 | 0.56 |
|  | Pto-SPL20-SNP43 | 3.25 | Pto-SPL15-SNP65 | 1.32 | 0.34 | -4.23 |
|  | Pto-SPL20-SNP43 | 3.25 | Pto-SPL15-SNP82 | 1.46 | 2.50 | -2.21 |
|  | Pto-SPL20-SNP43 | 3.25 | Pto-SPL15-SNP110 | 0 | 0.21 | -3.04 |
